# Exosome-mediated ferroptosis in the tumor microenvironment: from molecular mechanisms to clinical application

**DOI:** 10.1038/s41420-025-02484-y

**Published:** 2025-05-06

**Authors:** Na Liu, Tianqing Wu, Guohu Han, Minbin Chen

**Affiliations:** 1 Department of Radiotherapy and Oncology, Affiliated Kunshan Hospital of Jiangsu University, Kunshan, China; 2 XJTLU Wisdom Lake Academy of Pharmacy, Suzhou, Jiangsu Province China; 3 Department of Oncology, Jingjiang People’s Hospital Affiliated with Yangzhou University, Jingjiang, China

**Keywords:** Cancer, Cancer microenvironment

## Abstract

Ferroptosis in the tumor microenvironment (TME) plays a crucial role in the development, metastasis, immune escape, and drug resistance of various types of cancer. A better understanding of ferroptosis in the TME could illuminate novel aspects of this process and promote the development of targeted therapies. Compelling evidence indicates that exosomes are key mediators in regulating the TME. In this respect, it is now understood that exosomes can deliver biologically functional molecules to recipient cells, influencing cancer progression by reprogramming the metabolism of cancer cells and their surrounding stromal cells through ferroptosis. In this review, we focus on the role of exosomes in the TME and describe how they contribute to tumor reprogramming, immunosuppression, and the formation of pre-metastatic niches through ferroptosis. In addition, we highlight exosome-mediated ferroptosis as a potential target for cancer therapy and discuss strategies employing exosomes in ferroptosis treatment. Finally, we outline the current applications and challenges of targeted exosome-mediated ferroptosis therapy in tumor immunotherapy and chemotherapy. Our aim is to advance research on the link between exosomes and ferroptosis in the TME, and we pose questions to guide future studies in this area.

## Facts

- The exchange of exosomal cargo between cancer cells and their surrounding stromal cells in TME and defining the functional outcome of such exchange on tumor growth, metastasis and drug resistance by mediate the sensitivity of cells to ferroptosis.
- Exosome-mediated ferroptosis is a targeted pathway for cancer therapy.
- Exosomes derived from different tissues or cells inhibit ferroptosis through different pathways, which may be due to the differences in exosome contents.
- The combination of exosomes and ferroptosis has excellent potential for clinical application in cancer therapy.

## Open questions

- Is there cross-talk between nucleic acids, proteins, lipids and other substances contained in the same cell-derived exosomes in the regulation of ferroptosis?
- There are exosomes secreted by various cells in TME. How do exosomes from different cell sources choose recipient cells to regulate cancer cell ferroptosis? In addition, how can we identify which exosomes are friends and which are enemies?
- Does the amount and contents of exosomes secreted by cancer cells and stromal cells differ in the early, middle and late stages of cancer development, and do they change with the progression of cancer?
- How can engineered exosomes coated with ferroptosis inducers be modified to efficiently and specifically reach the recipient cancer cells to induce ferroptosis of cancer cells without triggering ferroptosis of antitumor immune cells? Moreover, how can appropriately designed exosomes be selected in different tumors?

## Introduction

The tumor microenvironment (TME) is a complex environmental network composed of tumor cells, stromal cells, extracellular matrix (ECM), surrounding blood vessels, and cell-secreted products such as exosomes and cytokines. Stromal cells, in particular, exhibit significant diversity, including immune cells, fibroblasts, adipogenic cells, and mesenchymal stromal cells [1]. Both cellular and noncellular components of the TME are extensively involved in cancer progression [2]. Consequently, therapeutic strategies targeting the TME have become a promising approach for cancer treatment [3].

Extracellular vesicles (EVs) represent a diverse group of membrane-bound vesicles released by cells into the extracellular environment [4]. They are widely found in cell culture supernatants and various bodily fluids (blood, saliva, cerebrospinal fluid, amniotic fluid, urine, etc.) and contain various cellular components, including proteins, DNA, messenger RNA (mRNA), circular RNA (circRNA), microRNAs (miRNAs), long intergenic noncoding RNA (LincRNA), lipids, and more [4]. Based on their origin and size, EVs can be categorized into four main subsets: ectosomes, exosomes, apoptotic bodies, and oncosomes [5]. Exosomes, originating from endosomes and with diameters ranging from ~40 to 160 nm, have become one of the most extensively studied EV types in recent years [6]. Acting as important mediators of cell-to-cell communication, exosomes modulate the biological functions of recipient cells by facilitating the exchange of various substances and can participate in both health and disease, impacting various aspects of cell biology [6, 7].

Exosomes secreted by tumor cells and stromal cells are key mediators of intercellular communication within the tumor microenvironment. They play a role in regulating the immune system, energy metabolism, cell metastasis, and drug resistance [8–10]. Recent studies have increasingly demonstrated that exosome-mediated cross-talk within the TME influences ferroptosis [11, 12]. Ferroptosis is a form of metabolism-regulated cell death caused by an imbalance between oxidation and antioxidant systems within cells [13]. Within the TME, exosomes released by various cells can either promote or inhibit ferroptosis depending on their contents [14, 15]. This exosome-mediated regulation of ferroptosis, shaping tumor reprogramming, the immunosuppressive microenvironment, and pre-metastatic niche formation within the TME, offers promising potential targets for future anticancer therapies.

## Exosomes and ferroptosis

### Biogenesis, characteristics, and functions of exosome

**Fig. 1:**
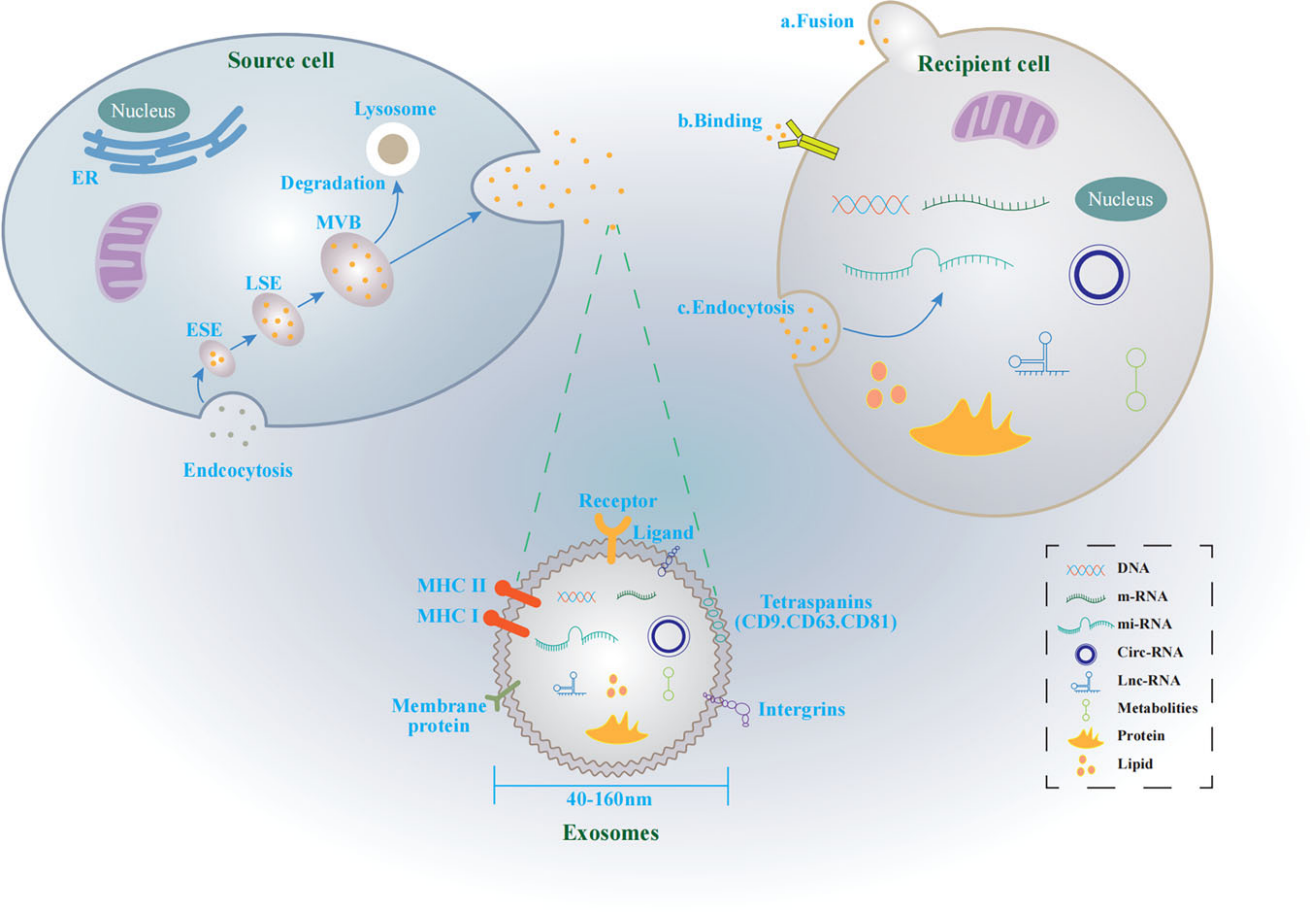
The biogenesis and characteristics of exosomes. Exosomes are secreted by source cells into the intercellular microenvironment through the multivesicular bodies (MVBs). Exosomes can transfer biologically functional molecules to recipient cells through three ways, including **a** exosomes directly fuse with the recipient cells membrane and release their contents; **b** intercellular signaling through receptor–ligand binding; **c** recipient cells phagocytose exosomes.

In 1983, Johnstone and colleagues first coined the term “exosomes” while studying vesicles in sheep reticulocytes [16]. Over the past decades, exosome research has progressed significantly, leading to a comprehensive understanding of their secretion process, structure, function, characteristics, and extraction methods. Exosome production occurs in three steps: first, the cell membrane invaginates to form early endosomes; second, early endosomes mature into multivesicular bodies (MVBs); finally, MVBs fuse with the cell membrane and release exosomes into the extracellular space [17]. These exosomes carry a significant portion of their parent cell’s material, including proteins, nucleic acids, lipids, and metabolites [6, 17] (Fig. 1). Besides, exosomes play a crucial role in cellular communication by transferring substances like proteins and nucleic acids to neighboring and distant cells, thereby altering the biological behavior of recipient cells [6, 18]. Exosomes interact with target cells through three primary mechanisms: (1) receptor–ligand interaction, where surface molecules on exosomes bind to specific receptors on target cells, triggering signaling pathways; (2) endocytosis or phagocytosis, where the target cell engulfs the exosome and incorporates its contents; and (3) membrane fusion, where the exosome membrane merges with the target cell membrane, directly delivering its contents [6] (Fig. 1).

Like cancer cells, exosomes exhibit significant heterogeneity. The morphology, diameter, contents, and functions of exosomes can vary depending on their cell of origin [6]. This heterogeneity can stem from differences in the source organ or tissue, as well as variations in the cellular environment, leading to unique functional roles [19–22]. For example, exosomes secreted by mesenchymal stem cells (MSCs) yield anti-inflammatory effects and can promote vascular and tissue regeneration. In contrast, cancer cell-derived exosomes often contribute to tumor metastasis, immune suppression, and drug resistance [19–22]. Functionally, exosomes can induce diverse effects on recipient cells due to the expression of cell surface receptors. This functional heterogeneity means that exosomes might induce cell survival, cell death, immune suppression, or immune activation, depending on the target cell type [18, 23, 24]. The complexity and heterogeneity of exosome function underscore the need for improved precision in modern medicine for anticancer therapy.

In general, the functions of exosomes in cancers can be summarized as follows: (I) intercellular information transmission function; (II) shaping the immune microenvironment; (III) The construction of pre-metastatic niche; (IV) Tumor angiogenesis [6].

### Overview of ferroptotic cell death

Ferroptosis, an iron-dependent form of regulated cell death characterized by excessive lipid peroxidation, has emerged as a promising target for cancer treatment [13, 25]. Morphologically, ferroptotic cells exhibit mitochondrial shrinkage and reduced mitochondrial cristae. Mechanistically, ferroptosis results from disruptions in iron metabolism, fatty acid synthesis and peroxidation, and imbalances within amino acid-based antioxidant systems [13, 25]. Under physiological conditions, a dynamic equilibrium exists between ferroptosis execution and defense systems [25]. However, when cellular metabolism is dysregulated (e.g., through excessive iron accumulation or hyperactive mitochondrial metabolism), this oxidative/antioxidant balance is disrupted. This leads to the generation of significant amounts of reactive oxygen species (ROS), triggering the peroxidation of specific membrane lipids and ultimately inducing ferroptosis [25]. Thus, the occurrence of ferroptosis depends on the interplay between ferroptosis execution and defense systems.

Ferroptosis execution system: Ferroptosis is an iron-dependent form of cell death characterized by the peroxidation of membrane lipids containing polyunsaturated fatty acids (PUFAs). This process is driven by iron-catalyzed Fenton reactions and enzymes with iron as a cofactor, such as lipoxygenases (LOXs) [25].

Specifically, acetyl-CoA is converted to malonyl-CoA by acetyl-CoA carboxylase (ACC), initiating the generation of polyunsaturated fatty acids (PUFAs). Then, under the action of long-chain acyl-CoA synthetase 4 (ACSL4) and lysophosphatidylcholine acyltransferase 3 (LPCAT3), PUFAs are integrated into phospholipids to form PUFA-PLs (polyunsaturated fatty acid phospholipids) [25]. In the presence of iron-dependent lipoxygenase and reactive oxygen species, PUFA-PLs undergo peroxidation, generating PUFA-PL-OOH (polyunsaturated fatty acid phospholipid hydroperoxide) and triggering ferroptosis [25, 26]. During this process, the transferrin receptor (TFR1) delivers Fe^3+^ into the cell. This Fe^3+^ is localized to endosomes and reduced to Fe^2+^ by STEAP3. Divalent metal transporter 1 (DMT1) then releases Fe^2+^ from endosomes into the labile iron pool, where it participates in ROS generation and lipoxygenase activation. Excess iron is stored in ferritin, which can be degraded through autophagy, releasing labile Fe^2+^ and further promoting lipid peroxidation. Additionally, mitochondria contribute to the ROS pool that drives ferroptosis [27–30].

The ferroptosis defense system operates through two key mechanisms: (1) enhancing cellular antioxidant capacity and (2) scavenging lipid peroxides. These functions primarily rely on the activity of glutathione peroxidase 4 (GPX4) [31]. System Xc-, a crucial regulator of ferroptosis, is a cystine/glutamate antiporter composed of two subunits: SLC7A11 and SLC3A2 [31]. This system imports cystine, a precursor for the antioxidant glutathione (GSH), while exporting glutamate [31]. GSH serves as a cofactor for GPX4, enabling it to reduce harmful lipid peroxides back to normal phospholipids and prevent ferroptosis [31]. Consequently, GPX4 is considered a critical target in ferroptosis therapy. Recent research has identified additional ferroptosis defense systems beyond the SLC7A11-GSH-GPX4 axis, including ferroptosis suppressor protein 1 (FSP1)/ubiquinone CoQ10/NAD(P)H, dihydroorotate dehydrogenase (DHODH)/CoQH2, cyclase GCH1/tetrahydrobiopterin (BH4), and ACSL3/MUFA-PL pathways [32]. More detailed information on ferroptosis is available in recent comprehensive reviews [13, 25].

**Fig. 2:**
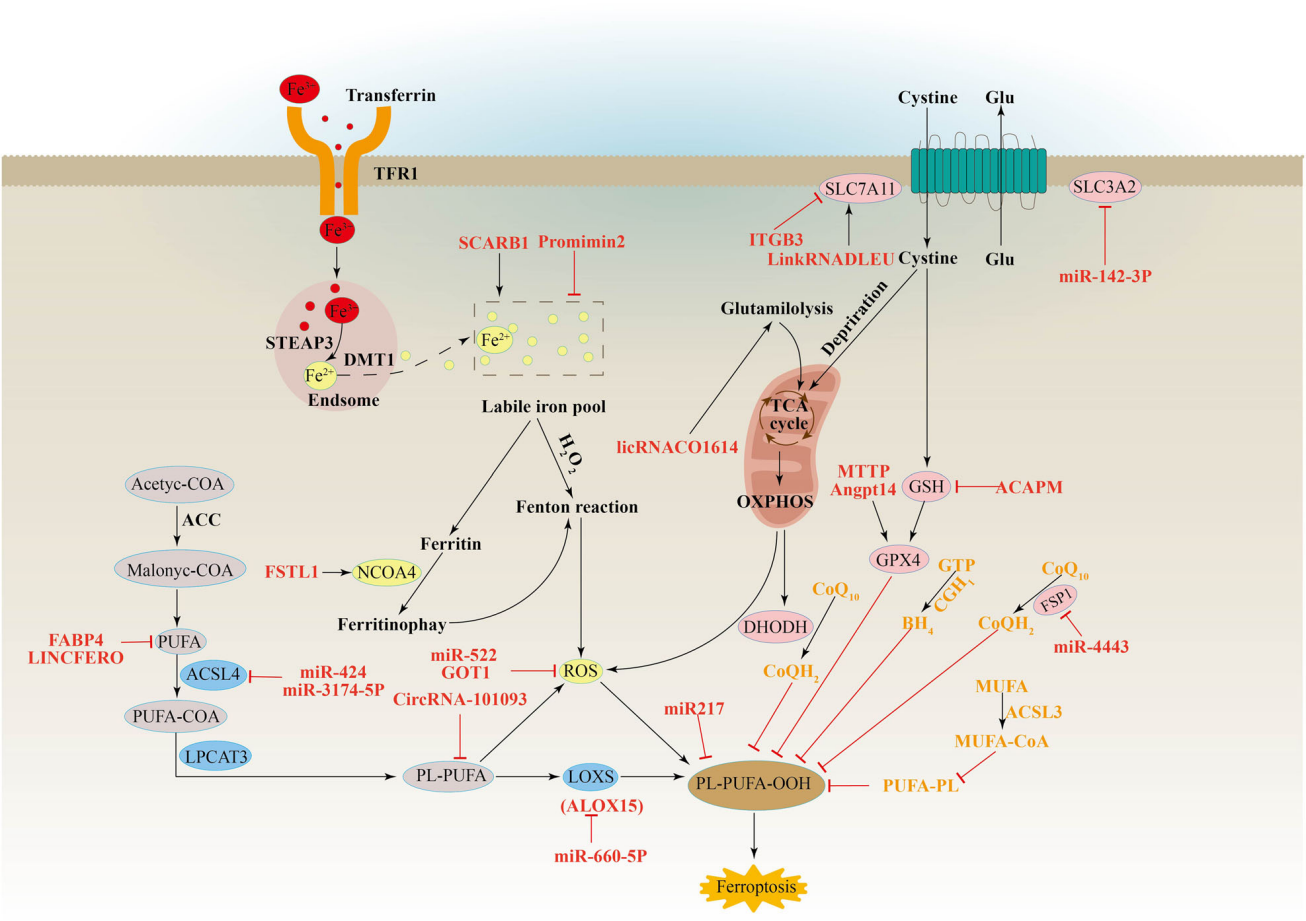
Mechanisms through which different sources of exosomes regulate ferroptosis in TME. Exosome-derived protein, miRNA, lincRNA, and circRNA regulate multiple intracellular ferroptosis pathways to promote or inhibit ferroptosis. (Black arrows indicate positive effects. Red perpendicular bars indicate negative effects.) The ferroptosis diagram quotes the review we have published [151].

Exosomes released by various cells within the tumor microenvironment contain proteins, mRNA, circRNA, miRNA, and lincRNA. These components influence ferroptosis by targeting recipient cells and regulating pathways like iron metabolism, fatty acid metabolism, GSH-GPX4, and FSP1/CoQ10/NAD(P)H. By influencing ferroptosis, exosomes can contribute to the reshaping of the TME, potentially creating a niche favorable for cancer invasion and metastasis (Fig. 2). We will explore this concept in detail in the following sections.

## Exosome-mediated ferroptosis in TME

**Fig. 3:**
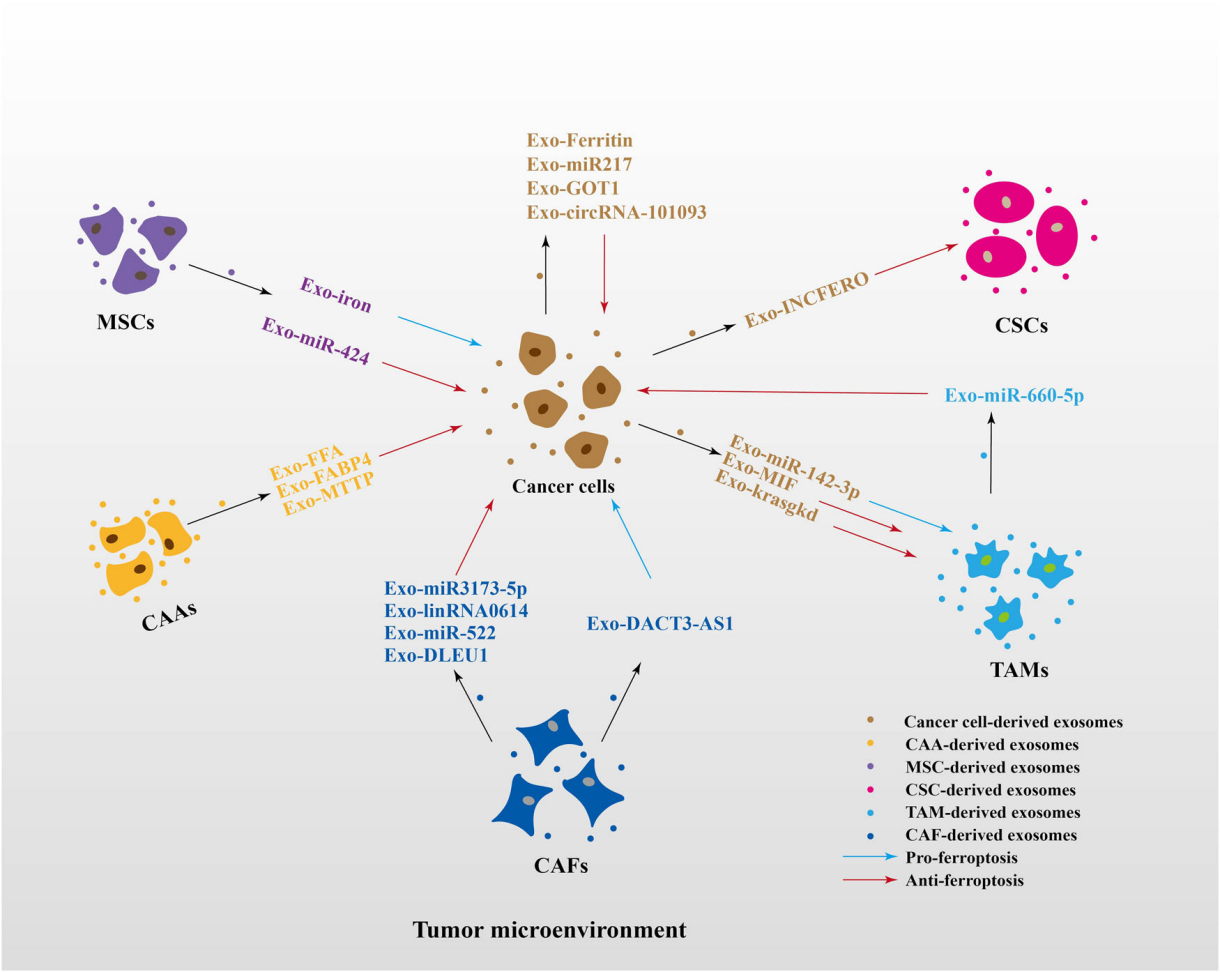
The exosome-mediated ferroptosis in the tumor microenvironment. Exosome-mediated ferroptosis occurs in cancer cells and their surrounding stromal cells in the TME. Stromal cells ferroptosis is affected by exosomes derived from cancer cells, and acts as a feedback loop to resistant ferroptosis in cancer cells or to provide a new niche required for cancer progression.

Exosomes transport various bioactive molecules (e.g., proteins, nucleic acids, lipids) from donor cells to recipient cells, leading to target cell reprogramming and epigenetic modifications [33, 34]. Numerous studies have demonstrated that exosomes regulate ferroptosis by influencing key pathways such as iron metabolism, lipid metabolism, and amino acid metabolism. This regulation affects cancer cell behavior and drug sensitivity [35, 36]. Within the tumor microenvironment, cancer cell-derived exosomes protect stromal cells from ferroptosis and contribute to stromal cell reprogramming (phenotypic alterations), immunosuppression, and metabolic remodeling—processes that favor cancer progression. Conversely, stromal cell-derived exosomes can promote ferroptosis resistance in cancer cells, leading to recurrence, metastasis, and drug resistance [35–37]. Therefore, exosome-mediated communication between tumor cells and stromal cells is a bidirectional process (Fig. 3). Importantly, ferroptosis signaling within the TME also influences the secretion of exosomes from both tumor and stromal cells [15, 38]. This interaction creates a vicious cycle that perpetuates cancer progression. A deeper understanding of the pathological mechanisms involving exosomes and ferroptosis in cancer will facilitate the development of novel targeted therapies for malignant tumors, enabling more precise and effective treatment strategies.

### Cancer cell

Similar to other cells, cancer cells release exosomes to facilitate material transport and communication with their environment [7]. Research indicates that cancer cell-derived exosomes dynamically interact with recipient cells, such as cancer-associated fibroblasts (CAFs), cancer-associated adipocytes (CAAs), and immune cells, within the tumor microenvironment. This interaction contributes to both cancer cell plasticity and stromal cell reprogramming [39–43].

Cancer cells can resist ferroptosis by dynamically upregulating iron export pathways. Exosomes act as transporters, facilitating the export of iron from cancer cells [15, 38], thereby limiting intracellular iron accumulation and inhibiting ferroptosis [15, 38]. For example, studies have shown that treating breast cancer cells with ferroptosis inducers like RSL3 and erastin increased the expression of prominin-2, a ferroptosis stress response protein. This promoted the formation of ferritin-containing multivesicular bodies (MVBs) and exosomes, which subsequently exported iron from the cells and inhibited ferroptosis [15, 38]. In this context, exosomes can be considered analogous to “iron-laden lorries”. Furthermore, studies have demonstrated that exosomes released by lung cancer cells can harbor circRNA-101093. This specific circRNA promotes cancer cell resistance to ferroptosis by reducing arachidonic acid (AA) levels and hindering its incorporation into the plasma membrane through the action of fatty acid-binding protein 3 (FABP3) [36]. In addition, exosomes released from cancer cells can contain aspartate transaminase 1 (GOT1) and miR-217. These factors contribute to cancer cell survival and progression by reducing ROS accumulation and inhibiting ferroptosis [35, 44]. However, miR-144-3p, derived from osteosarcoma cell exosomes, negatively regulates ZEB1 expression, influencing iron metabolism and ROS levels to induce ferroptosis in cancer cells [45]. These findings suggest a potentially “binary” effect of cancer cell-derived exosomes on ferroptosis. Overall, these results highlight the role of exosomes in mediating cancer cell ferroptosis. Targeting exosomes secreted by cancer cells may represent a promising new approach to cancer treatment.

It has been established that cancer cell-derived exosomes influence ferroptosis sensitivity both within cancer cells and in surrounding stromal cells. In gastric cancer (GC) cells, exosome-transmitted lncFERO reduces the formation of PUFAs, inhibiting ferroptosis in gastric cancer stem cells (CSCs). This promotes their stemness, contributing to tumor recurrence and drug resistance [46]. Similarly, exosome-released miR-142-3p from hepatocellular carcinoma (HCC) cells targets SLC3A2 expression, inducing ferroptosis in M1 macrophages. This leads to immunosuppression and accelerates HCC progression [47]. Collectively, these studies demonstrate how cancer cell-derived exosomes transmit ferroptosis signals to neighboring stromal cells, modulating their ferroptosis sensitivity and ultimately influencing cancer development.

Using ferroptosis agonists to induce exosome release from tumor cells can have a dual effect. On the one hand, the proteins and nucleic acids carried by these exosomes may inhibit key ferroptosis pathways, protecting cancer cells from this form of cell death. On the other hand, these exosomes can transmit ferroptosis-related signals throughout the tumor microenvironment, potentially inducing cell death in recipient cells. Further, these recipient cells may then produce additional exosomes that contribute to ferroptosis in other cells via paracrine signaling. Therefore, the efficacy of ferroptosis therapy depends on the balance between these two opposing effects. Researchers have recently proposed therapeutic strategies aimed at blocking cancer cell exosome secretion to enhance ferroptosis in cancer cells. However, a crucial question remains: how can we distinguish between exosomes that are beneficial for cancer treatment and those that promote cancer progression?

### Cancer-associated adipocytes

Adipocytes, widely distributed throughout the human body, primarily function to store and release free fatty acids (FFAs) to support local and systemic metabolic requirements [48]. Research has established a strong link between obesity and poor prognosis in various cancers, including breast cancer and colorectal cancer [49–51]. Studies indicate that adipocytes and tumor cells engage in bidirectional communication via exosomes. For instance, oleic acid, a metabolic product secreted by adipocytes, has been shown to inhibit ferroptosis in breast cancer cells [52]. Conversely, cancer cell-derived exosomes promote phenotypic changes in adipocytes, leading to the formation of cancer-associated adipocytes [43, 53]. CAAs promote a proinflammatory and invasive phenotype in tumor cells through the exchange of cytokines and lipids, leading to metabolic reprogramming [43, 53]. In obese individuals, lipid accumulation enhances the cancer-promoting effects of CAAs within the tumor microenvironment. This may explain the clinically observed poor prognosis in obese breast cancer patients [53, 54].

Exosomes released by cancer-associated adipocytes contribute to cancer progression by influencing recipient cancer cells through the following mechanisms: (I) Adipokine secretion: CAAs secrete adipocyte-derived factors such as leptin, adiponectin, TNF-α, and IL-6, which promote cancer progression; (II) Fatty acids and related proteins [55, 56]. Myeloma cells take up these FAs, and high levels of FA uptake lead to increased lipid peroxidation and ferroptosis induction [57, 58]. Conversely, CAA-derived exosomes can release FABP4 and MTTP, which regulate fatty acid production and antioxidant capacity, thereby inhibiting lipid peroxidation [49, 54, 56].

In conclusion, CAAs influence cancer progression by regulating ferroptosis through metabolic reprogramming mediated by exosomes. The molecules involved in this bidirectional metabolic exchange between CAAs and cancer cells hold promise as potential therapeutic targets for cancer treatment.

### Cancer-associated fibroblasts

Cancer-associated fibroblasts, the most abundant stromal cell type within the tumor microenvironment, play a crucial role in tumor growth, metastasis, immunosuppression, and drug resistance by secreting various factors, including exosomes and inflammatory cytokines [59–61].

Numerous studies have demonstrated the critical role of CAFs in regulating ferroptosis and consequently, tumor progression [14, 62]. For example, a study showed that miR-3173-5p, derived from CAF exosomes, acted as a “sponge” for ACSL4, thereby inhibiting ferroptosis in cancer cells upon uptake [62].

Furthermore, miR-522, secreted by CAF-derived exosomes in chemotherapy-treated gastric cancer cells, was found to reduce ROS accumulation and inhibit ferroptosis, contributing to the development of drug resistance [14]. Interestingly, long noncoding RNAs (lncRNAs) associated with CAF exosomes also play a role in ferroptosis regulation. One study identified that CAFs upregulate the level of lncRNA DLEU1, which promotes the expression of SLC7A11. Subsequently, SLC7A11 promotes cysteine uptake for glutathione (GSH) synthesis, thereby inhibiting ferroptosis in glioblastoma cells [63]. Another study reported that the CAF-specific lncRNA LINC01614 enhances glutamine uptake in lung adenocarcinoma [64]. Glutamine is a critical factor in ferroptosis; insufficient glutamine or blockade of its breakdown prevents ROS accumulation, leading to the inhibition of lipid peroxidation and ferroptosis [65]. These findings further emphasize the importance of CAF-derived exosomes in modulating the ferroptosis sensitivity of cancer cells.

CAFs-derived exosomes can interact with not only cancer cells but also immune cells, exerting immunosuppressive effects [23]. In gastric cancer, these exosomes contribute to ferroptosis induction in natural killer (NK) cells, thereby disrupting the antitumor immune response and promoting tumor progression and immune escape. This occurs through the export of iron into the tumor microenvironment via the upregulation of iron regulatory genes ferroportin1 and hephaestin in CAF-derived exosomes. This process increases the labile iron pool within NK cells, ultimately triggering ferroptosis [23]. Notably, follistatin-like protein 1 (FSTL1) released from CAF-derived exosomes enhances NCOA4 expression, and ferritinophagy mediated by NCOA4 is essential for CAF-mediated ferroptosis in NK cells [23].

In conclusion, CAF-derived exosomes harbor various molecules, including proteins, microRNAs, and lncRNAs, that can be transferred to cancer cells or other stromal cells. These molecules influence key ferroptosis pathways in recipient cells, thereby contributing to cancer progression.

### Tumor-associated macrophages

Tumor-associated macrophages (TAMs) are a major immune cell type within the tumor microenvironment, exhibiting high plasticity and heterogeneity [66, 67]. In response to environmental stimuli, TAMs undergo polarization. Activated macrophages are generally categorized into two phenotypes: M1 (anti-tumor) and M2 (pro-tumor) [68, 69]. The M1 phenotype produces ROS and inflammatory factors that directly kill tumor cells. It also helps activate other immune cells to participate in the antitumor immune response. In contrast, the M2 phenotype produces anti-inflammatory factors, growth factors, and angiogenesis factors that promote tumor cell proliferation, metastasis, and immunosuppression. M2 macrophages inhibit the function of T cells, NK cells, and other immune cells involved in the immunosuppressive response [68–71].

Studies have demonstrated that exosomes can influence the polarization of tumor-associated macrophages [72–74]. For instance, exosomes derived from pancreatic and colorectal cancer cells release microRNAs (miRNAs) or proteins that are taken up by macrophages, promoting their M2 polarization and facilitating an immunosuppressive response that fosters cancer progression [72–74]. However, melanoma-derived exosomes harbor miR-125b-5p, which targets lysosomal acid lipase A (LIPA) and promotes M1 polarization of TAMs. This contributes to antitumor immune responses, disrupts the tumor microenvironment, and induces ferroptosis in cancer cells [75]. Therefore, exosomes secreted by cancer cells can influence the M1 or M2 phenotype of TAMs within tumors. Furthermore, ferroptosis signaling also appears to regulate macrophage polarization and participate in reshaping the tumor immune microenvironment [76]. Studies have shown that inducing ferroptosis in TAMs isolated from hepatocellular carcinoma (HCC) tissues, leading to the production of reactive oxygen species, can promote their polarization to the M1 phenotype. This, in turn, exerts an immune-activating effect and inhibits HCC progression [76]. Importantly, inflammatory cytokines such as TNF-α, IL-6, and IL-1β, released by macrophages, influence iron and lipid metabolism, potentially creating conditions conducive to ferroptosis [77]. This highlights the interconnectedness between ferroptosis occurrence and the state of macrophages.

TAMs can inhibit ferroptosis in cancer cells by secreting exosomes that deliver specific signaling molecules [37]. For example, TAM-secreted factors IL-4 and IL-13 activate STAT6, which induces the expression of miR-660-5p. This microRNA (miRNA) is then transported via exosomes to cervical cancer cells, where it inhibits the expression of ALOX15, thereby suppressing ferroptosis [37]. Another study demonstrated that macrophages undergoing ferroptosis during asbestos phagocytosis produce extracellular vesicles (EVs). These EVs can be internalized by mesothelial cells, leading to the production of ferroptosis-dependent extracellular vesicles (FedEVs). The uptake of FedEVs by recipient mesothelial cells results in genomic damage and the development of malignant mesothelioma. This suggests that FedEVs contribute to asbestos-induced mesothelioma development by transporting iron, a key mutagenic mediator [78]. Therefore, exosome-mediated ferroptosis plays a significant role in intercellular communication between macrophages and cancer cells within the tumor microenvironment.

### Mesenchymal stromal cells

Mesenchymal stem cells are adult stem cells with multi-lineage differentiation potential and self-renewal capacity. Found throughout the body in tissues such as bone marrow and adipose, MSCs play a role in the healing and repair of various injuries, including those to the lung, heart, and spinal cord [79, 80]. However, numerous studies have also shown that MSCs contribute to tumor progression and drug resistance [81–83]. Interestingly, Tumor cell-secreted factors can reprogram MSCs into cancer-associated MSCs (CA-MSCs), which exhibit pro-tumorigenic functions. CA-MSCs can influence cancer progression by inhibiting the immune response, promoting angiogenesis, and enhancing epithelial-mesenchymal transition [84]. Exosomes play a key role in the communication between MSCs and cancer cells. For instance, studies have shown that miR-142-3p, carried by exosomes derived from bone marrow mesenchymal stem cells (BM-MSCs), increased the number of cancer stem cells [85]. In addition, exosomes derived from BM-MSCs promoted proliferation, invasion, and chemotherapy resistance in acute myeloid leukemia cells by upregulating S100A4 [86]. These findings suggest that MSCs exosomes can increase the metastatic potential of tumor cells by promoting their motility and invasiveness, and may contribute to the formation of metastatic niches. However, contradictory evidence also exists. For example, bone marrow mesenchymal stem cell-derived exosomal miR-425-5p inhibited the proliferation, apoptosis, invasion, and migration of acute myeloid leukemia cells by targeting Wilms tumor 1-associated protein (WTAP) [87]. Further investigation into the complex interactions between MSCs and other components of the tumor microenvironment is necessary. A comprehensive assessment of the effects of MSCs on cancer initiation, growth, and spread could help identify novel therapeutic targets.

In recent years, numerous studies have demonstrated that MSC-derived exosomes can deliver proteins and nucleic acids to target cells, thereby inhibiting ferroptosis. These exosomes have been shown to play a protective role in various conditions such as liver injury, inflammatory bowel disease, ischemia-reperfusion injury, and heart damage caused by chemotherapeutic drug toxicity [88–92]. However, the role of exosome-mediated ferroptosis in cell communication between MSCs and cancer cells, and its potential impact on tumor progression, remains relatively understudied. While limited research exists, some scholars have proposed a link between MSC-derived exosomes and ferroptosis in cancer progression. On the one hand, MSCs can deliver iron to cancer cells, leading to iron accumulation and increased sensitivity to ferroptosis inducers [93]. Conversely, other studies have reported that MSC-derived exosomes can inhibit ferroptosis. For example, MSC-derived exosomes have been shown to deliver miR-424 to lung cancer cells, downregulating ACSL4 expression and consequently inhibiting ferroptosis [94]. Although the current body of research exploring the role of MSC-derived exosomes in regulating ferroptosis and its impact on cancer is limited, the existing findings are promising and offer valuable insights into the potential application of MSC-derived exosomes in cancer therapy.

## Exosome-mediated ferroptosis in cancer progression

**Fig. 4:**
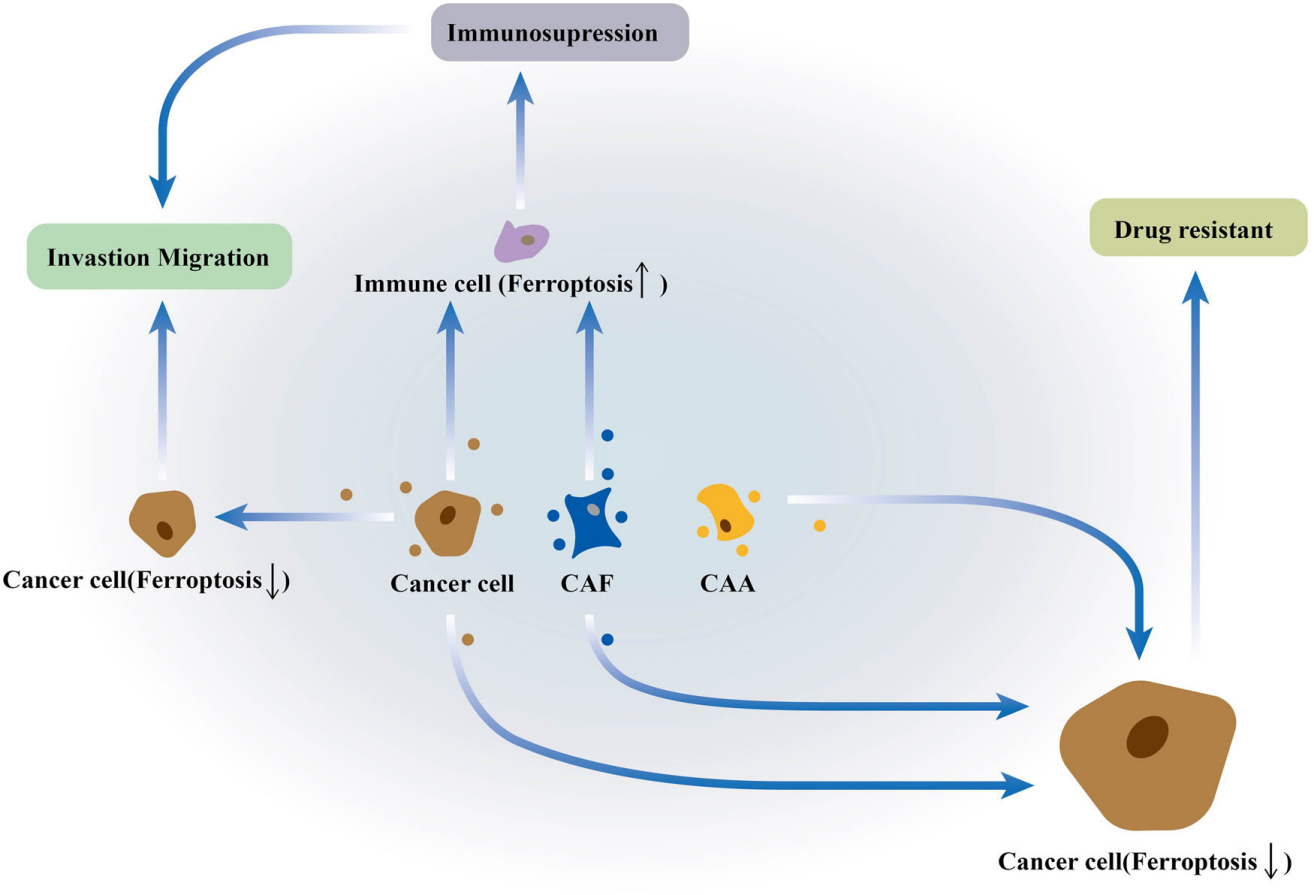
The exosome-mediated ferroptosis in the cancer progression. Exosomes are vitally involved in tumor metastasis, immunosuppression, and drug resistance by ferroptosis.

A growing body of research demonstrates the role of exosomes in remodeling the tumor microenvironment. Exosomes play a crucial part in cell phenotype transformation, immunosuppression, angiogenesis, extracellular matrix remodeling, proliferation, invasion, metastasis, and drug resistance [33, 82, 95]. Importantly, exosomes facilitate cell-cell communication within the TME, delivering proteins, nucleic acids, and metabolites. This intercellular communication contributes to cancer progression and is partially achieved through the regulation of ferroptosis (Fig. 4) [96].

### Tumor metastasis

The metastasis of cancer cells, which involves their invasion of vital organs and tissues, presents a significant challenge in clinical treatment and is a major cause of cancer-related deaths [97]. Compelling evidence suggests that exosomes play a role in the metastatic process [98, 99]. Within the tumor microenvironment, exosomes derived from cancer cells or stromal cells contribute to angiogenesis, immune suppression, metabolic reprogramming, and other processes that create an environment conducive to metastasis [99–103]. Importantly, the formation of a pre-metastatic niche is essential for supporting the colonization of distant sites and promoting metastasis. Exosomes play a key role in establishing this pre-metastatic niche [34, 104, 105]. Through exosomal communication, tumor cells actively interact with neighboring cells and their local microenvironment, mediating ferroptosis in both cancer cells and stromal cells [106]. Next, we expound on the effect of exosome-mediated ferroptosis on cancer metastasis.

Exosomes derived from nasopharyngeal carcinoma (NPC) release macrophage migration inhibitory factor (MIF). Macrophages can absorb MIF, which not only inhibits macrophage ferroptosis but also promotes their M2 polarization [107]. Chen and colleagues demonstrated that macrophages can internalize NPC-secreted exosomes, leading to the regulation of iron metabolism and ROS levels. This promotes ferroptosis in M1 macrophages while upregulating CYP1B1 to reduce phagocytosis by M2 macrophages, resulting in immunosuppression that aids NPC metastasis [96]. In addition, exosomes released from hepatocellular carcinoma cells deliver miR-142-3p, which regulates the expression of key ferroptosis proteins like TfR1 and GPX4. This induces ferroptosis in M1 macrophages and promotes HCC invasion [47]. Collectively, exosomes derived from NPC and HCC exert immunosuppressive effects by mediating ferroptosis in immune cells, creating favorable conditions for cancer cell metastasis. Interestingly, platelets also communicate with tumor cells through exosomes [108]. EVs released from platelets in nasopharyngeal carcinoma patients can increase integrin β3 (ITGB3) expression in NPC cells [108]. Intriguingly, ITGB3 inhibits SLC7A11 expression, enhancing ferroptosis resistance in cancer cells and promoting distant metastasis via the bloodstream [108]. The above studies overlap in their assertion non-tumor cells that take up exosomes secreted by cancer cells are involved in establishing a pre-metastatic niche, thereby contributing to the metastatic capacity of the tumor. Exosomes clearly act as key facilitators in the development of tumor pre-metastatic niches.

### Tumor immunosuppression

Ferroptosis plays a multifaceted role in tumor immunity. On the one hand, immunosuppressive cells such as M2 macrophages, T regulatory (Treg) cells, and tumor-infiltrating neutrophils (TINs) rely on GPX4 and other factors to prevent ferroptosis and maintain their pro-tumor activity. Therefore, ferroptosis inducers can lead to the inactivation of these cells, disrupting their immunosuppressive effects, activating antitumor immunity, and hindering cancer progression [109–111]. On the other hand, inducing ferroptosis can also inadvertently cause the inactivation of antitumor immune cells such as CD8 + T cells, NK cells, and dendritic cells, leading to immune deficiency and promoting cancer progression [110–114]. This highlights that the induction of ferroptosis triggers ferroptosis in both malignant tumor cells and immune cells, potentially impairing antitumor immunity. Interestingly, ferroptosis induction in colorectal cancer has been shown to enhance CD8 + T-cell function, which may be beneficial for tumor immunotherapy [115]. This suggests that ferroptosis can potentially improve the tumor’s immunosuppressive microenvironment and increase tumor sensitivity to immunotherapy [115]. These findings illustrate the complex regulatory network between ferroptosis and immunity.

Recent research has increasingly highlighted the significant role of exosomes in shaping the tumor immunosuppressive microenvironment by mediating ferroptosis in immune cells [72, 107]. For instance, exosomes derived from NPC and CRC cells have been shown to prevent ferroptosis in macrophages and promote their M2 polarization, thereby contributing to an immunosuppressive response that fosters cancer progression [72, 107]. Similarly, exosomal miR-142-3p derived from hepatocellular carcinoma cells has been found to induce ferroptosis in M1 macrophages, leading to reduced antitumor immunity and promoting liver cancer invasion [47]. In gastric cancer, studies have demonstrated that exosomes derived from cancer-associated fibroblasts can induce ferroptosis in NK cells by increasing their labile iron pool. This ultimately disrupts the antitumor immune response and promotes tumor progression and immune escape [23]. These findings collectively suggest that exosome-mediated ferroptosis-induced immunosuppression may represent a potential driver of tumor progression, highlighting the complex interplay between ferroptosis and the TME.

### Tumor drug resistance

The development of tumor drug resistance remains a major hurdle in achieving effective cancer treatment outcomes in clinical practice. This significantly limits the selection and use of available anticancer drugs [116]. Currently, several well-described mechanisms contribute to tumor drug resistance, including increased drug efflux and decreased drug uptake, target mutations, altered signaling pathways, enhanced DNA damage repair, and phenotypic transitions (cellular plasticity) [117–119]. A substantial number of clinical trials have demonstrated that the development of novel drugs targeting resistance mechanisms and the combinatorial use of multiple targeted therapies are the primary strategies to overcome the challenge of drug resistance [118, 120]. Therefore, gaining a deeper understanding of these mechanisms and implementing measures to counteract them can significantly improve treatment outcomes and prognoses for cancer patients.

Exosomes and ferroptosis have recently emerged as critical players in the development and progression of drug resistance. Interestingly, numerous recent studies have revealed that exosome-mediated ferroptosis can not only contribute to resistance against chemotherapeutic drugs such as oxaliplatin, cisplatin, paclitaxel, and gemcitabine but also influence the sensitivity of cancer cells to radiotherapy [14, 62, 121, 122].

The uptake of CAFs-derived exosomal miR-522 by cancer cells confers resistance to ferroptosis by targeting arachidonic acid lipoxygenase 15 (ALOX15) and blocking lipid ROS accumulation. This ultimately leads to decreased sensitivity of gastric cancer cells to paclitaxel and cisplatin [14]. Interestingly, another study demonstrated that CAFs can secrete the long noncoding RNA disheveled-binding antagonist of beta catenin3 antisense1 (DACT3-AS1). DACT3-AS1 promotes ferroptosis by targeting the miR-181a-5p/sirtuin 1 (SIRT1) axis, thereby inhibiting cell proliferation, migration, and invasion, and sensitizing gastric cancer cells to oxaliplatin [121]. Importantly, DACT3-AS1 expression is downregulated in gastric cancer and is associated with poor patient prognosis; however, the mechanism underlying this downregulation remains unclear [121].

Treatment of pancreatic cancer with gemcitabine can induce an adaptive resistance response. Specifically, gemcitabine treatment stimulates the release of miR-3173-5p by exosomes secreted from CAFs. This miR-3173-5p then “sponges” ACSL4, thereby inhibiting ferroptosis and promoting gemcitabine resistance in pancreatic cancer cells [62]. Recent studies suggest that the presence of medium-chain acyl-CoA dehydrogenase (ACADM) in exosomes derived from pancreatic cancer cells can be used as a marker for gemcitabine treatment sensitivity. Mechanistically, ACADM promotes the consumption of unsaturated fatty acids and subsequent GSH depletion, ultimately reducing intracellular lipid peroxides and reactive oxygen species levels, leading to ferroptosis inhibition and gemcitabine resistance. Importantly, knocking down ACADM in pancreatic cancer cells could improve their sensitivity to gemcitabine treatment [123].

Cisplatin is a widely used chemotherapeutic drug for lung cancer treatment. It exerts its anticancer effect by inducing ferroptosis, a cell death pathway mediated by the protein FSP1 [124]. Studies have shown that exosomes derived from cisplatin-resistant lung cancer cells exhibit high levels of miR-4443 [124]. When co-cultured with cisplatin-sensitive cell lines, these exosomes can deliver miR-4443 to the sensitive cells. This delivery of miR-4443 by exosomes promotes the development of cisplatin resistance in lung cancer by inhibiting FSP1 m6A modification and subsequently ferroptosis [124]. Therefore, strategies targeting exosome secretion or specifically targeting miR-4443 hold promise for overcoming cisplatin resistance in lung cancer.

In addition to cancer-associated fibroblasts and cancer cell-derived exosomes, adipocyte-derived exosomes have also been shown to influence chemotherapeutic efficacy through ferroptosis [49]. A study demonstrated that microprotein translocator protein (MTTP) released from adipocyte exosomes regulates the PRAP1/ZEB1 axis, leading to the upregulation of GPX4 and downregulation of ACSL4. This, in turn, inhibits lipid ROS production and PUFA levels, ultimately suppressing ferroptosis and promoting oxaliplatin resistance in colorectal cancer [49]. Notably, the study also found that chemotherapy drug toxicity can further promote MTTP expression and secretion in adipocytes [49]. This creates a vicious cycle, where adipocyte-derived exosomes contribute to drug resistance, and the resulting chemotherapeutic stress further increases MTTP expression, exacerbating the resistance. Targeting adipocyte MTTP may, therefore, represent a promising strategy to overcome clinical drug resistance in colorectal cancer patients [49].

Hypoxia, a defining feature of the tumor microenvironment, is a major contributor to radiotherapy resistance [125, 126]. Recent studies have shown that exosomes derived from lung cancer cells under hypoxic conditions exhibit significantly higher levels of the protein angiopoietin-like 4 (ANGPTL4) compared to those under normoxic conditions. Importantly, high levels of ANGPTL4 can significantly enhance the resistance of non-small cell lung cancer (NSCLC) cells to radiation [122]. Mechanistically, ANGPTL4 upregulates key ferroptosis proteins such as GPX4, SLC11A7, and FTH4, leading to reduced lipid peroxidation accumulation and inhibited iron-dependent cell death, ultimately promoting radioprotection [122].

These findings collectively highlight the contribution of cancer and stromal cell-derived exosomes to drug resistance by regulating ferroptosis. Targeting exosome-mediated ferroptosis, therefore, emerges as a potential novel therapeutic strategy to overcome drug resistance in cancer patients.

## The potential clinical application of exosome-mediated ferroptosis in cancer

Exosomes within the TME carry specific bioactive substances that mediate bidirectional transfer between cancer cells and stromal cells. This exchange plays a role in regulating ferroptosis in recipient cells, ultimately affecting cancer progression. Exosome-mediated ferroptosis has two potential clinical applications in cancer, one diagnostic and the other therapeutic.

### Liquid biopsy for cancer diagnosis

Exosome-mediated ferroptosis represents an emerging research direction in cancer diagnostics. Exosomal biomolecules (such as miRNAs, proteins, lipids, etc.) associated with ferroptosis may serve as non-invasive biomarkers. As intercellular communication carriers, exosomes can transport signaling molecules related to ferroptosis (such as miRNAs, GPX4, ACSL4, iron metabolites, etc.), reflecting tumor progression, prognosis, and treatment sensitivity [14, 45, 49, 62, 127]. Exosomal ferroptosis-related miRNAs can be categorized into two groups: miRNAs that promote ferroptosis of tumor cells, for example, miR-144-3p [45], and those that inhibit tumor cell ferroptosis, for example, miR-424, miR-522, and miR-3174-5P, etc., discussed in the above chapter [14, 62, 94].The expression levels of these exosomal miRNAs may serve as an indicator of cancer progression, prognosis, and sensitivity to treatment. Similarly, exosomal ferroptosis-related protein markers (GPX4, ACSL4) and lipid peroxidation products (4HNE) may also reflect tumor cell ferroptosis activity and serve as potential tumor diagnostic tools [15, 128, 129]. Therefore, the expression profiles of exosome-derived miRNAs, proteins, and lipids associated with ferroptosis may be related to tumor progression and facilitate the differentiation of cancer types, stages, or therapeutic responses [14, 15, 45, 49, 62, 127–129]. Exosome-mediated ferroptosis-related markers utilized as liquid biopsies offer advantages, including high specificity, non-invasive sampling, and dynamic monitoring [130, 131]. However, several challenges remain: First of all, significant heterogeneity exists in exosomal markers across different cancer types and individuals. Besides, the complex bidirectional regulatory mechanisms regulating ferroptosis and exosome interactions warrant further clarification. Exosome-mediated ferroptosis signaling (especially miRNAs) exhibits substantial potential in cancer diagnosis. With the development of AI(Artificial Intelligence)-assisted diagnosis, this field may provide new tools for early detection and precision medicine. Future studies should integrate multiple omics. Large-scale cohort studies are required to validate the sensitivity and specificity of a diagnostic model based on exosomal miRNA, protein, and lipidomic data.

### Exosome-based ferroptosis therapy in cancer

The combination of exosome biology and ferroptosis presents novel therapeutic strategies for cancer treatment, termed exosome-based ferroptosis therapy. Current research in this field has focused on three primary aspects: (I) Targeted inhibition of proteins, nucleic acids, metabolites released by exosomes that contribute to cancer ferroptosis resistance; (II) Construction of engineered exosomes based ferroptosis therapy (FT): the ferroptosis inducer was encapsulated in the exosomes, and the exosome surface was modified to improve the biocompatibility and targeting specificity to achieve the maximum effect to induce ferroptosis; (III) Combining engineered exosomes for ferroptosis therapy with chemotherapeutic agents, immunodrugs, photodynamic agents, etc.

As discussed, exosomes derived from cancer cells or stromal cells can release proteins, miRNAs, lncRNAs, and other molecules that contribute to the remodeling of the tumor microenvironment. These exosomal components can render cancer cells resistant to ferroptosis by influencing iron, lipid, and amino acid metabolism. Therefore, exosomes play a crucial role in cancer cell desensitization to ferroptosis, and blocking their endogenous production emerges as a promising strategy to induce ferroptosis and enhance chemotherapeutic efficacy.

In gastric cancer, treatment with cisplatin and paclitaxel has been shown to stimulate the secretion of exosomal miR-522 by cancer-associated fibroblasts. This miR-522 downregulates ALOX15 and reduces ROS levels, ultimately inhibiting ferroptosis. Notably, knockdown of miR-522 significantly increased cell death. Consequently, inhibiting the exosomal secretion of miR-522 by CAFs represents a novel therapeutic approach for gastric cancer [14]. Exosomes can also function as siderophores, facilitating the transfer of iron out of cells, thereby promoting ferroptosis resistance [15]. To counter this resistance, Wang and colleagues designed a nanoparticle that inhibits exosome secretion and restores intracellular iron levels, ultimately reversing ferroptosis resistance [15, 132]. Similar strategies targeting exosome secretion have been explored in lung cancer, bladder cancer, pancreatic cancer, and liver cancer, aiming to prevent the release of molecules like circRNA-101093, miR-217, GOT1, and miR-142-3p, which contribute to ferroptosis resistance [35, 36, 44, 47].

In conclusion, exosome-mediated ferroptosis resistance, originating from cancer cells or stromal cells, plays a critical role in cancer development and drug resistance. By analyzing exosomal contents, we can identify specific proteins, miRNAs, lncRNAs, and metabolites that promote ferroptosis resistance in cells. Targeting these factors may offer a direct and effective strategy to improve the clinical outcomes of cancer patients. However, our current understanding of the cellular and molecular mechanisms governing exosome biogenesis, release, uptake, and function remains limited. Further research is needed to explore the intricate link between exosomes and ferroptosis. Future studies should focus on in-depth pathway analysis to achieve a comprehensive molecular understanding of how exosomes contribute to the inhibition of ferroptosis.

Exosomes have gained significant traction as a potential drug delivery system in contemporary cancer research [133]. However, their therapeutic application is currently hindered by limitations such as inefficient delivery and inadequate tumor targeting [133]. To address these limitations, various techniques can be employed to modify the surface proteins or contents of exosomes under controlled conditions. These commonly used modification strategies include: biological modification (target peptide), immune modification (antibody), physical modification (magnetic particles), and chemical modification (sodium bicarbonate) [133, 134]. Importantly, the development and application of engineered exosomes offer promising solutions to overcome the current limitations of cancer chemotherapeutic agents.

In this section, we collectively refer to therapeutic strategies that induce ferroptosis in cancer cells as ferroptosis therapies (FTs). However, ferroptosis inducers like erastin face limitations in clinical application due to their poor water solubility and high renal toxicity [135]. One promising approach involves the use of folate-modified exosomes as carriers for ferroptosis inducers. This strategy is exemplified by the development of erastin-loaded folate-modified exosomes (erastin@FA-exo), which significantly enhance cellular uptake of erastin compared to the free drug [45]. In breast cancer cells, erastin@FA-exo treatment effectively depletes glutathione, promotes reactive oxygen species generation, and ultimately induces ferroptosis, leading to inhibited proliferation and migration [135]. Another study utilized M1 macrophage-derived exosomes (M1-exo) loaded with the ferroptosis inducer RSL3, creating “RSL3-exo” complexes. This strategy enhanced the tumor distribution of RSL3, leading to reduced GSH and GPX4 expression and increased ROS-mediated ferroptosis [127]. Based on these studies, we propose that the synergistic combination of exosome properties and ferroptosis induction holds great promise for the future development of cancer therapy.

Remarkably, exosomes derived from mesenchymal stem cells have shown huge potential as safe carriers of antitumor drugs for the treatment of osteosarcoma. By modifying MSC-derived exosomes with the bone-targeting peptide SDSSD and encapsulating capreomycin (CAP), researchers have constructed a nano-platform that induces ferroptosis in osteosarcoma (OS) cells. This effect is achieved by promoting the accumulation of reactive oxygen species, Fe^2+^ aggregation, and lipid peroxidation [136]. Engineered exosomes have diverse applications beyond this example. Recently, researchers have proposed the development of Er/RB@ExosCD47 [137]. Erastin (Er) is a ferroptosis inducer, while Rose Bengal (RB) is a photosensitizer that generates high ROS concentrations during photodynamic therapy (PDT). The synergistic delivery of Erastin and RB to tumor tissues has the potential to induce ferroptosis with enhanced efficacy [137]. Macrophage-mediated phagocytosis represents a significant barrier to effective drug delivery using exosomes. However, incorporating anti-phagocytic molecules, such as CD47, on the exosome surface can prolong their circulation half-life and improve bioavailability within target tissues [138]. Taking this into consideration, Du’s group employed transfection technology to incorporate CD47 into exosomes, protecting them from macrophage phagocytosis. In addition, the ferroptosis inducer Erastin and photosensitizer Rose Bengal were encapsulated into the exosomes using ultrasound, resulting in the drug-loaded construct Er/RB@ExosCD47 [137]. Er/RB@ExosCD47 was demonstrated to efficiently and specifically induce ferroptosis in hepatocellular carcinoma cells under light exposure [137].

These findings strongly suggest that the synergistic combination of exosomes and ferroptosis holds immense potential for clinical translation in cancer therapy. Currently, preclinical studies exploring exosomes loaded with anticancer drugs that target ferroptosis pathways are actively underway. This research paves the way for the development of novel anticancer drugs targeting diverse ferroptosis pathways in the future.

Preclinical studies of engineered exosomes for cancer treatment have yielded promising results in recent years. A notable example involves the use of engineered exosomes conjugated with magnetic nanoparticles to simultaneously dismantle the ferroptosis defense axis in glioblastoma treatment [139]. Importantly, researchers are exploring the potential of engineered exosomes to enhance the efficacy of other antitumor therapies, including chemotherapy and immunotherapy.

#### Chemotherapy

The clinical application of chemotherapeutic drugs is often hampered by their limitations, including high systemic toxicity and insufficient tumor targeting [140]. However, numerous studies have demonstrated that encapsulating chemotherapeutic drugs like doxorubicin (DOX), docetaxel, and paclitaxel (PTX) within engineered exosomes can leverage the unique advantages of these exosomes—their inherent tumor targeting and intracellular delivery capabilities. This approach has been shown to achieve more potent antitumor activity while reducing systemic toxicity [139, 141–143]. Notably, the applications of exosomes extend beyond overcoming limitations of existing drugs and can also address the challenge of drug resistance.

Sorafenib (SORA) exerts its anticancer effect by inducing ferroptosis. However, ferroptosis inhibitor proteins such as CPX4, DHODH, and HSPB1 are often highly expressed in SORA-resistant cancer cells, hindering the drug’s efficacy. Therefore, strategies to induce ferroptosis offer a promising approach to overcome SORA resistance [144, 145]. Studies have shown that miR-654-5p enhances SORA-induced ferroptosis by binding to HSPB1 and reducing its protein levels [144]. To improve the in vivo stability and delivery efficiency of miR-654-5p, Sun et al. constructed an engineered small extracellular vesicle (sEV) using human adipose-derived mesenchymal stem cells as a vector for miR-654-5p, termed m654-sEV [144]. Notably, the combination of sorafenib and m654-sEV effectively suppressed HSPB1 expression and significantly inhibited tumor growth compared to sorafenib treatment alone [144]. Similarly, Li et al.‘s research group constructed engineered exosomes loaded with multiplex small interfering RNA (multi-siRNA) targeting the ferroptosis suppressor genes GPX4 and DHODH. This strategy aimed to silence the expression of these genes and induce ferroptosis in SORA-resistant cells, thereby overcoming drug resistance [145]. Their study demonstrated that the combination of multi-siRNA-containing exosomes and sorafenib significantly inhibited tumor growth compared to the control group [145]. In conclusion, combining engineered exosomes with sorafenib not only effectively addresses the issue of SORA resistance but also significantly enhances the drug’s anticancer activity.

#### Immunotherapy

An increasing body of evidence demonstrates that natural exosomes derived from tumor cells or stromal cells contribute to shaping a tumor immunosuppressive microenvironment through immune cell suppression and immune surveillance escape [18, 146]. This TME significantly hinders the effectiveness of immunotherapy [44]. Consequently, utilizing engineered exosomes to remodel the immunosuppressive TME has emerged as a promising strategy to enhance immunotherapy efficacy [127, 147].

The introduction of immune checkpoint inhibitor therapy has revolutionized cancer treatment; however, some tumors develop resistance, leading to poor clinical outcomes [147]. Tumor-derived exosomes not only impair the function of dendritic cells and T cells but also secrete PD-L1, contributing to the failure of PD-1/PD-L1 checkpoint inhibitor therapy [147, 148]. Studies by Wang et al. demonstrated that inhibiting the secretion of tumor-derived exosomes could improve immunotherapy efficacy. They engineered a nanounit complex combining an exosome inhibitor (GW4869) and a ferroptosis inducer (Fe^3+^) with amphiphilic hyaluronic acid, significantly enhancing the antitumor immune response [149]. However, depleting exosomal PD-L1 does not influence its expression on tumor cell surfaces, where it can still potently suppress immunotherapy [149]. Notably, a study comparing the combined use of the engineered nanounit and an anti-PD-L1 antibody with the nanounit alone revealed that the combination not only further elevated T-cell activation and proliferation but also hindered cancer metastasis [149]. These findings suggest that targeted anti-exosome therapy can synergize with systemic anti-PD-L1 therapy to elicit a robust systemic immune response against highly metastatic tumors [149]. Furthermore, Dai et al. constructed a photodiagnostic metal-phenolic network (PFG mpn) by encapsulating a semiconductor polymer with a ferroptosis-inducing agent (Fe^3+^) and an exosome inhibitor (GW4869). This novel platform combines photothermal therapy (PTT) with anti-exosomal PD-L1, leading to a synergistic effect that enhances ferroptosis and exerts significant antitumor activity [150].

Beyond utilizing exosome inhibitors to reshape the immune microenvironment, researchers are exploring alternative strategies. One approach involves loading M1 macrophage-derived exosomes (M1-exo) with the ferroptosis inducer RSL3, creating “RSL3-exo” complexes. This strategy enhances the intratumoral distribution of RSL3 while reducing the expression of glutathione (GSH) and the antioxidant enzyme GPX4 [127]. Furthermore, RSL3-exo promotes the immune response by synergistically increasing ROS production, ferroptosis-related protein expression, and ultimately, elimination of the immunosuppressive tumor microenvironment [49]. Notably, RSL3-exo treatment significantly increased the proportion of M1 macrophages while decreasing M2 macrophages in tumors compared to controls and RSL3 alone, further highlighting its potential for TME modulation [127]. Fibroblast activation protein-α (FAP)-positive cancer-associated fibroblasts and macrophages represent potential targets for improving the tumor immunosuppressive microenvironment [11, 127]. For example, Hu et al. developed exosome-like nanovesicles derived from FAP-genetically engineered tumor cells (eNVs-FAP), demonstrating promising antitumor effects in various tumor models [11, 127]. Mechanistically, eNVs-FAP suppresses tumor growth through a multifaceted approach: (1) reducing immunosuppressive cell populations like MDSCs, M2 macrophages, and Tregs, (2) eliminating FAP-positive CAFs within the tumor microenvironment, and (3) reprogramming the TME to promote immune activation and tumor ferroptosis [11, 127]. This strategy using exosome-based immunotherapy targeting ferroptosis holds immense potential for treating malignant metastatic tumors. Future research should prioritize identifying novel cargo molecules with functionalities that can be harnessed to design effective therapeutic strategies.

## Conclusions and perspectives

As discussed earlier, this review focused on the exchange of exosomal cargo between cancer cells and their surrounding stromal cells within the tumor microenvironment. We further explored the functional consequences of this exchange on tumor growth, metastasis, and drug resistance by examining its role in mediating cellular sensitivity to ferroptosis. These findings highlight exosome-mediated ferroptosis as a promising targeted pathway for cancer therapy. Interestingly, exosomes derived from different tissues or cell types exhibit diverse mechanisms for inhibiting ferroptosis, potentially due to variations in their cargo composition. Therefore, a deeper understanding of the exosomal protein, nucleic acid, and metabolite content and its influence on ferroptosis regulation is of significant importance.

While current research on the regulation of cancer ferroptosis by exosomes remains limited, existing findings are highly promising. To fully grasp the significance of exosomes in ferroptosis regulation, several intriguing questions require further investigation: (I) Do exosomal cargo molecules within the same cell-derived exosome interact (synergistically or antagonistically) to regulate ferroptosis? (II) How do exosomes from diverse cell sources within the TME specifically target recipient cells for cancer cell ferroptosis regulation? How can we distinguish “friendly” from “enemy” exosomes in this context? (III) Do the quantity and content of exosomes secreted by cancer and stromal cells vary across the early, middle, and late stages of cancer development, and do these changes correlate with cancer progression? (IV) Since external factors like chemotherapy or hypoxia can alter exosome secretion and content, does this render them unsuitable for cancer diagnosis or prognosis indicators? (V) How can engineered exosomes loaded with ferroptosis inducers be modified to specifically target cancer cells and induce ferroptosis without affecting antitumor immune cells? In addition, how can we tailor these exosomes for optimal efficacy in different tumor types?

Preclinical research on exosome-mediated ferroptosis has primarily relied on cell-based experiments. To translate this promising approach into clinical reality, further investigations are necessary. Long-term monitoring platforms and robust in vivo models are crucial to assess the biosafety and efficacy of exosome-based ferroptosis therapy. Large-scale, multi-center, and long-term clinical trials are urgently needed to bridge the gap between preclinical findings and clinical application of engineered exosomes. Beyond delving deeper into the mechanisms of exosome-mediated ferroptosis, future research should prioritize developing novel and effective strategies for engineering exosomes. The successful clinical translation of exosome-based ferroptosis therapy remains the ultimate goal.

## References

[1] Arner E, Rathmell J. Metabolic programming and immune suppression in the tumor microenvironment. Cancer Cell. 2023;41:421–33. 10.1016/j.ccell.2023.01.009. 36801000 10.1016/j.ccell.2023.01.009 PMC10023409

[2] de Visser K, Joyce J. The evolving tumor microenvironment: from cancer initiation to metastatic outgrowth. Cancer Cell. 2023;41:374–403. 10.1016/j.ccell.2023.02.016. 36917948 10.1016/j.ccell.2023.02.016

[3] Pitt J, Marabelle A, Eggermont A, Soria J, Kroemer G, Zitvogel L. Targeting the tumor microenvironment: removing obstruction to anticancer immune responses and immunotherapy. Ann Oncol. 2016;27:1482–92. 10.1093/annonc/mdw168. 27069014 10.1093/annonc/mdw168

[4] Dixson A, Dawson T, Di Vizio D, Weaver A. Context-specific regulation of extracellular vesicle biogenesis and cargo selection. Nat Rev Mol Cell Biol. 2023;24:454–76. 10.1038/s41580-023-00576-0. 36765164 10.1038/s41580-023-00576-0 PMC10330318

[5] Li S, Man Q, Gao X, Lin H, Wang J, Su F, et al. Tissue-derived extracellular vesicles in cancers and non-cancer diseases: present and future. J Extracell Vesicles. 2021;10:e12175. 10.1002/jev2.12175. 34918479 10.1002/jev2.12175 PMC8678102

[6] Kalluri R, LeBleu V. The biology function and biomedical applications of exosomes. Science 2020;367:eaau6977. 10.1126/science.aau6977. 10.1126/science.aau6977 PMC7717626 32029601

[7] Becker A, Thakur B, Weiss J, Kim H, Peinado H, Lyden D. Extracellular vesicles in cancer: cell-to-cell mediators of metastasis. Cancer Cell. 2016;30:836–48. 10.1016/j.ccell.2016.10.009. 27960084 10.1016/j.ccell.2016.10.009 PMC5157696

[8] Li I, Nabet B. Exosomes in the tumor microenvironment as mediators of cancer therapy resistance. Mol Cancer. 2019;18:32. 10.1186/s12943-019-0975-5. 30823926 10.1186/s12943-019-0975-5 PMC6397467

[9] Huang L, Rong Y, Tang X, Yi K, Qi P, Hou J, et al. Engineered exosomes as an in situ DC-primed vaccine to boost antitumor immunity in breast cancer. Mol Cancer. 2022;21:45. 10.1186/s12943-022-01515-x. 35148751 10.1186/s12943-022-01515-x PMC8831689

[10] Li C, Teixeira A, Zhu H, Ten Dijke P. Cancer associated-fibroblast-derived exosomes in cancer progression. Mol Cancer. 2021;20:154. 10.1186/s12943-021-01463-y. 34852849 10.1186/s12943-021-01463-y PMC8638446

[11] Hu S, Ma J, Su C, Chen Y, Shu Y, Qi Z, et al. Engineered exosome-like nanovesicles suppress tumor growth by reprogramming tumor microenvironment and promoting tumor ferroptosis. Acta Biomater. 2021;135:567–81. 10.1016/j.actbio.2021.09.003. 34506976 10.1016/j.actbio.2021.09.003

[12] Yuan Y, Mei Z, Qu Z, Li G, Yu S, Liu Y, et al. Exosomes secreted from cardiomyocytes suppress the sensitivity of tumor ferroptosis in ischemic heart failure. Signal Transduct Target Ther. 2023;8:121. 10.1038/s41392-023-01336-4. 36967385 10.1038/s41392-023-01336-4 PMC10040407

[13] Jiang X, Stockwell B, Conrad M. Ferroptosis: mechanisms, biology and role in disease. Nat Rev Mol Cell Biol. 2021;22:266–82. 10.1038/s41580-020-00324-8. 33495651 10.1038/s41580-020-00324-8 PMC8142022

[14] Zhang H, Deng T, Liu R, Ning T, Yang H, Liu D, et al. CAF secreted miR-522 suppresses ferroptosis and promotes acquired chemo-resistance in gastric cancer. Mol Cancer. 2020;19:43. 10.1186/s12943-020-01168-8. 32106859 10.1186/s12943-020-01168-8 PMC7045485

[15] Brown C, Amante J, Chhoy P, Elaimy A, Liu H, Zhu L, et al. Prominin2 drives ferroptosis resistance by stimulating iron export. Dev Cell. 2019;51:575–86.e4. 10.1016/j.devcel.2019.10.007. 31735663 10.1016/j.devcel.2019.10.007 PMC8316835

[16] Pan B, Johnstone R. Fate of the transferrin receptor during maturation of sheep reticulocytes in vitro: selective externalization of the receptor. Cell. 1983;33:967–78. 10.1016/0092-8674(83)90040-5. 6307529 10.1016/0092-8674(83)90040-5

[17] Pegtel D, Gould S. Exosomes. Annu Rev Biochem. 2019;88:487–514. 10.1146/annurev-biochem-013118-111902. 31220978 10.1146/annurev-biochem-013118-111902

[18] Zhang L, Yu D. Exosomes in cancer development, metastasis, and immunity. Biochim et Biophys Acta Rev Cancer. 2019;1871:455–68. 10.1016/j.bbcan.2019.04.004. 10.1016/j.bbcan.2019.04.004 PMC6542596 31047959

[19] Khayambashi P, Iyer J, Pillai S, Upadhyay A, Zhang Y, Tran S. Hydrogel encapsulation of mesenchymal stem cells and their derived exosomes for tissue engineering. Int J Mol Sci. 2021;22:684. 10.3390/ijms22020684. 10.3390/ijms22020684 PMC7827932 33445616

[20] An Y, Lin S, Tan X, Zhu S, Nie F, Zhen Y, et al. Exosomes from adipose-derived stem cells and application to skin wound healing. Cell Prolif. 2021;54:e12993 10.1111/cpr.12993. 33458899 10.1111/cpr.12993 PMC7941238

[21] Xu Z, Chen Y, Ma L, Chen Y, Liu J, Guo Y, et al. Role of exosomal non-coding RNAs from tumor cells and tumor-associated macrophages in the tumor microenvironment. Mol Ther. 2022;30:3133–54. 10.1016/j.ymthe.2022.01.046. 35405312 10.1016/j.ymthe.2022.01.046 PMC9552915

[22] Li W, Li C, Zhou T, Liu X, Liu X, Li X, et al. Role of exosomal proteins in cancer diagnosis. Mol Cancer. 2017;16:145. 10.1186/s12943-017-0706-8. 28851367 10.1186/s12943-017-0706-8 PMC5576100

[23] Yao L, Hou J, Wu X, Lu Y, Jin Z, Yu Z, et al. Cancer-associated fibroblasts impair the cytotoxic function of NK cells in gastric cancer by inducing ferroptosis via iron regulation. Redox Biol. 2023;67:102923. 10.1016/j.redox.2023.102923. 37832398 10.1016/j.redox.2023.102923 PMC10582581

[24] Kok V, Yu C. Cancer-derived exosomes: their role in cancer biology and biomarker development. Int J Nanomed. 2020;15:8019–36. 10.2147/ijn.S272378. 10.2147/IJN.S272378 PMC7585279 33116515

[25] Stockwell B. Ferroptosis turns 10: emerging mechanisms, physiological functions, and therapeutic applications. Cell. 2022;185:2401–21. 10.1016/j.cell.2022.06.003. 35803244 10.1016/j.cell.2022.06.003 PMC9273022

[26] Li D, Li Y. The interaction between ferroptosis and lipid metabolism in cancer. Signal Transduct Target Ther. 2020;5:108. 10.1038/s41392-020-00216-5. 32606298 10.1038/s41392-020-00216-5 PMC7327075

[27] Tang D, Chen X, Kang R, Kroemer G. Ferroptosis: molecular mechanisms and health implications. Cell Res. 2021;31:107–25. 10.1038/s41422-020-00441-1. 33268902 10.1038/s41422-020-00441-1 PMC8026611

[28] Zheng J, Conrad M. The metabolic underpinnings of ferroptosis. Cell Metab. 2020;32:920–37. 10.1016/j.cmet.2020.10.011. 33217331 10.1016/j.cmet.2020.10.011

[29] Fang X, Ardehali H, Min J, Wang F. The molecular and metabolic landscape of iron and ferroptosis in cardiovascular disease. Nat Rev Cardiol. 2023;20:7–23. 10.1038/s41569-022-00735-4. 35788564 10.1038/s41569-022-00735-4 PMC9252571

[30] Rochette L, Dogon G, Rigal E, Zeller M, Cottin Y, Vergely C. Lipid peroxidation and iron metabolism: two corner stones in the homeostasis control of ferroptosis. Int J Mol Sci. 2022;24:449. 10.3390/ijms24010449. 10.3390/ijms24010449 PMC9820499 36613888

[31] Chen X, Yu C, Kang R, Kroemer G, Tang D. Cellular degradation systems in ferroptosis. Cell Death Differ. 2021;28:1135–48. 10.1038/s41418-020-00728-1. 33462411 10.1038/s41418-020-00728-1 PMC8027807

[32] Lei G, Zhuang L, Gan B. Targeting ferroptosis as a vulnerability in cancer. Nat Rev Cancer. 2022;22:381–96. 10.1038/s41568-022-00459-0. 35338310 10.1038/s41568-022-00459-0 PMC10243716

[33] Dai J, Su Y, Zhong S, Cong L, Liu B, Yang J, et al. Exosomes: key players in cancer and potential therapeutic strategy. Signal Transduct Target Ther. 2020;5:145. 10.1038/s41392-020-00261-0. 32759948 10.1038/s41392-020-00261-0 PMC7406508

[34] Wortzel I, Dror S, Kenific C, Lyden D. Exosome-mediated metastasis: communication from a distance. Dev Cell. 2019;49:347–60. 10.1016/j.devcel.2019.04.011. 31063754 10.1016/j.devcel.2019.04.011

[35] Guo Y, Chen T, Liang X, Gou S, Xiong J, Cui J, et al. Tumor cell derived exosomal GOT1 suppresses tumor cell ferroptosis to accelerate pancreatic cancer progression by activating Nrf2/HO-1 axis via upregulating CCR2 expression. Cells. 2022;11:3893. 10.3390/cells11233893. 10.3390/cells11233893 PMC9735520 36497150

[36] Zhang X, Xu Y, Ma L, Yu K, Niu Y, Xu X, et al. Essential roles of exosome and circRNA_101093 on ferroptosis desensitization in lung adenocarcinoma. Cancer Commun. 2022;42:287–313. 10.1002/cac2.12275. 10.1002/cac2.12275 PMC9017758 35184419

[37] Luo Y, Chen Y, Jin H, Hou B, Li H, Li X, et al. The suppression of cervical cancer ferroptosis by macrophages: the attenuation of ALOX15 in cancer cells by macrophages-derived exosomes. Acta Pharmaceutica Sin B. 2023;13:2645–62. 10.1016/j.apsb.2023.03.025. 10.1016/j.apsb.2023.03.025 PMC10326300 37425043

[38] Strzyz P. Iron expulsion by exosomes drives ferroptosis resistance. Nat Rev Mol Cell Biol. 2020;21:4–5. 10.1038/s41580-019-0195-2. 31748716 10.1038/s41580-019-0195-2

[39] Cho J, Park H, Lim E, Lee K. Exosomes from breast cancer cells can convert adipose tissue-derived mesenchymal stem cells into myofibroblast-like cells. Int J Oncol. 2012;40:130–138. 10.3892/ijo.2011.1193. 10.3892/ijo.2011.1193 21904773

[40] Webber J, Steadman R, Mason M, Tabi Z, Clayton A. Cancer exosomes trigger fibroblast to myofibroblast differentiation. Cancer Res. 2010;70:9621–30. 10.1158/0008-5472.Can-10-1722. 21098712 10.1158/0008-5472.CAN-10-1722

[41] Abd Elmageed Z, Yang Y, Thomas R, Ranjan M, Mondal D, Moroz K, et al. Neoplastic reprogramming of patient-derived adipose stem cells by prostate cancer cell-associated exosomes. Stem Cells. 2014;32:983–97. 10.1002/stem.1619. 24715691 10.1002/stem.1619 PMC4184251

[42] Le M, Hamar P, Guo C, Basar E, Perdigão-Henriques R, Balaj L, et al. miR-200-containing extracellular vesicles promote breast cancer cell metastasis. J Clin Investig. 2014;124:5109–28. 10.1172/jci75695. 25401471 10.1172/JCI75695 PMC4348969

[43] Wang S, Xu M, Li X, Su X, Xiao X, Keating A, et al. Exosomes released by hepatocarcinoma cells endow adipocytes with tumor-promoting properties. J Hematol Oncol. 2018;11:82. 10.1186/s13045-018-0625-1. 29898759 10.1186/s13045-018-0625-1 PMC6001126

[44] Huang Z, Wang H, Ji Z. Bladder cancer tissue-derived exosomes suppress ferroptosis of T24 bladder cancer cells by transporting miR-217. Environ Mol Mutagen. 2023;64:39–49. 10.1002/em.22520. 36461670 10.1002/em.22520

[45] Jiang M, Jike Y, Liu K, Gan F, Zhang K, Xie M, et al. Exosome-mediated miR-144-3p promotes ferroptosis to inhibit osteosarcoma proliferation, migration, and invasion through regulating ZEB1. Mol Cancer. 2023;22:113. 10.1186/s12943-023-01804-z. 37461104 10.1186/s12943-023-01804-z PMC10351131

[46] Zhang H, Wang M, He Y, Deng T, Liu R, Wang W, et al. Chemotoxicity-induced exosomal lncFERO regulates ferroptosis and stemness in gastric cancer stem cells. Cell Death Dis. 2021;12:1116. 10.1038/s41419-021-04406-z. 34845198 10.1038/s41419-021-04406-z PMC8629982

[47] Hu Z, Zhang H, Liu W, Yin Y, Jiang J, Yan C, et al. Mechanism of HBV-positive liver cancer cell exosomal miR-142-3p by inducing ferroptosis of M1 macrophages to promote liver cancer progression. Transl Cancer Res. 2022;11:1173–87. 10.21037/tcr-22-96. 35706810 10.21037/tcr-22-96 PMC9189167

[48] Crewe C, Funcke J, Li S, Joffin N, Gliniak C, Ghaben A, et al. Extracellular vesicle-based interorgan transport of mitochondria from energetically stressed adipocytes. Cell Metab. 2021;33:1853–68.e11. 10.1016/j.cmet.2021.08.002. 34418352 10.1016/j.cmet.2021.08.002 PMC8429176

[49] Zhang Q, Deng T, Zhang H, Zuo D, Zhu Q, Bai M, et al. Adipocyte-derived exosomal MTTP suppresses ferroptosis and promotes chemoresistance in colorectal cancer. Adv Sci. 2022;9:e2203357. 10.1002/advs.202203357. 10.1002/advs.202203357 PMC9534973 35978266

[50] Lapeire L, Hendrix A, Lambein K, Van Bockstal M, Braems G, Van Den Broecke R, et al. Cancer-associated adipose tissue promotes breast cancer progression by paracrine oncostatin M and Jak/STAT3 signaling. Cancer Res. 2014;74:6806–19. 10.1158/0008-5472.Can-14-0160. 25252914 10.1158/0008-5472.CAN-14-0160

[51] Lazar I, Clement E, Dauvillier S, Milhas D, Ducoux-Petit M, LeGonidec S, et al. Adipocyte exosomes promote melanoma aggressiveness through fatty acid oxidation: a novel mechanism linking obesity and cancer. Cancer Res. 2016;76:4051–7. 10.1158/0008-5472.Can-16-0651. 27216185 10.1158/0008-5472.CAN-16-0651

[52] Xie Y, Wang B, Zhao Y, Tao Z, Wang Y, Chen G, et al. Mammary adipocytes protect triple-negative breast cancer cells from ferroptosis. J Hematol Oncol. 2022;15:72. 10.1186/s13045-022-01297-1. 35659320 10.1186/s13045-022-01297-1 PMC9164506

[53] Dirat B, Bochet L, Dabek M, Daviaud D, Dauvillier S, Majed B, et al. Cancer-associated adipocytes exhibit an activated phenotype and contribute to breast cancer invasion. Cancer Res. 2011;71:2455–65. 10.1158/0008-5472.Can-10-3323. 21459803 10.1158/0008-5472.CAN-10-3323

[54] Bouche C, Quail D. Fueling the tumor microenvironment with cancer-associated adipocytes. Cancer Res. 2023;83:1170–2. 10.1158/0008-5472.Can-23-0505. 37057599 10.1158/0008-5472.CAN-23-0505

[55] Wu Q, Li B, Li J, Sun S, Yuan J, Sun S. Cancer-associated adipocytes as immunomodulators in cancer. Biomark Res. 2021;9:2. 10.1186/s40364-020-00257-6. 33413697 10.1186/s40364-020-00257-6 PMC7792018

[56] Luis G, Godfroid A, Nishiumi S, Cimino J, Blacher S, Maquoi E, et al. Tumor resistance to ferroptosis driven by stearoyl-CoA desaturase-1 (SCD1) in cancer cells and fatty acid biding protein-4 (FABP4) in tumor microenvironment promote tumor recurrence. Redox Biol. 2021;43:102006. 10.1016/j.redox.2021.102006. 34030117 10.1016/j.redox.2021.102006 PMC8163990

[57] Panaroni C, Fulzele K, Mori T, Siu K, Onyewadume C, Maebius A, et al. Multiple myeloma cells induce lipolysis in adipocytes and uptake fatty acids through fatty acid transporter proteins. Blood. 2022;139:876–88. 10.1182/blood.2021013832. 34662370 10.1182/blood.2021013832 PMC8832479

[58] Shafat M, Oellerich T, Mohr S, Robinson S, Edwards D, Marlein C, et al. Leukemic blasts program bone marrow adipocytes to generate a protumoral microenvironment. Blood. 2017;129:1320–32. 10.1182/blood-2016-08-734798. 28049638 10.1182/blood-2016-08-734798

[59] Mhaidly R, Mechta-Grigoriou F. Role of cancer-associated fibroblast subpopulations in immune infiltration, as a new means of treatment in cancer. Immunol Rev. 2021;302:259–72. 10.1111/imr.12978. 34013544 10.1111/imr.12978 PMC8360036

[60] Ying F, Chan M, Lee T. Cancer-associated fibroblasts in hepatocellular carcinoma and cholangiocarcinoma. Cell Mol Gastroenterol Hepatol. 2023;15:985–99. 10.1016/j.jcmgh.2023.01.006. 36708970 10.1016/j.jcmgh.2023.01.006 PMC10040968

[61] Rimal R, Desai P, Daware R, Hosseinnejad A, Prakash J, Lammers T, et al. Cancer-associated fibroblasts: origin, function, imaging, and therapeutic targeting. Adv Drug Deliv Rev. 2022;189:114504. 10.1016/j.addr.2022.114504. 35998825 10.1016/j.addr.2022.114504

[62] Qi R, Bai Y, Li K, Liu N, Xu Y, Dal E, et al. Cancer-associated fibroblasts suppress ferroptosis and induce gemcitabine resistance in pancreatic cancer cells by secreting exosome-derived ACSL4-targeting miRNAs. Drug Resist Updates. 2023;68:100960. 10.1016/j.drup.2023.100960. 10.1016/j.drup.2023.100960 37003125

[63] Zhao J, Yang S, Lv C, Liu Y. Cancer-associated fibroblasts suppressed ferroptosis in glioblastoma via upregulating lncRNA DLEU1. Am J Physiol Cell Physiol. 2023;324:C1039–52. 10.1152/ajpcell.00454.2022. 36878845 10.1152/ajpcell.00454.2022

[64] Liu T, Han C, Fang P, Ma Z, Wang X, Chen H, et al. Cancer-associated fibroblast-specific lncRNA LINC01614 enhances glutamine uptake in lung adenocarcinoma. J Hematol Oncol. 2022;15:141. 10.1186/s13045-022-01359-4. 36209111 10.1186/s13045-022-01359-4 PMC9548164

[65] Gao M, Monian P, Quadri N, Ramasamy R, Jiang X. Glutaminolysis and transferrin regulate ferroptosis. Mol cell. 2015;59:298–308. 10.1016/j.molcel.2015.06.011. 26166707 10.1016/j.molcel.2015.06.011 PMC4506736

[66] Chamseddine A, Assi T, Mir O, Chouaib S. Modulating tumor-associated macrophages to enhance the efficacy of immune checkpoint inhibitors: a TAM-pting approach. Pharmacol Ther. 2022;231:107986. 10.1016/j.pharmthera.2021.107986. 34481812 10.1016/j.pharmthera.2021.107986

[67] Shapouri-Moghaddam A, Mohammadian S, Vazini H, Taghadosi M, Esmaeili S, Mardani F, et al. Macrophage plasticity, polarization, and function in health and disease. J Cell Physiol. 2018;233:6425–40. 10.1002/jcp.26429. 29319160 10.1002/jcp.26429 PMC13482560

[68] Gao J, Liang Y, Wang L. Shaping polarization of tumor-associated macrophages in cancer immunotherapy. Front Immunol. 2022;13:888713. 10.3389/fimmu.2022.888713. 35844605 10.3389/fimmu.2022.888713 PMC9280632

[69] Li M, Yang Y, Xiong L, Jiang P, Wang J, Li C. Metabolism, metabolites, and macrophages in cancer. J Hematol Oncol. 2023;16:80. 10.1186/s13045-023-01478-6. 37491279 10.1186/s13045-023-01478-6 PMC10367370

[70] Mantovani A, Marchesi F, Malesci A, Laghi L, Allavena P. Tumour-associated macrophages as treatment targets in oncology. Nat Rev Clin Oncol. 2017;14:399–416. 10.1038/nrclinonc.2016.217. 28117416 10.1038/nrclinonc.2016.217 PMC5480600

[71] DeNardo D, Ruffell B. Macrophages as regulators of tumour immunity and immunotherapy. Nat Rev Immunol. 2019;19:369–82. 10.1038/s41577-019-0127-6. 30718830 10.1038/s41577-019-0127-6 PMC7339861

[72] Dai E, Han L, Liu J, Xie Y, Kroemer G, Klionsky D, et al. Autophagy-dependent ferroptosis drives tumor-associated macrophage polarization via release and uptake of oncogenic KRAS protein. Autophagy. 2020;16:2069–83. 10.1080/15548627.2020.1714209. 31920150 10.1080/15548627.2020.1714209 PMC7595620

[73] Wang X, Luo G, Zhang K, Cao J, Huang C, Jiang T, et al. Hypoxic tumor-derived exosomal miR-301a mediates M2 macrophage polarization via PTEN/PI3Kγ to promote pancreatic cancer metastasis. Cancer Res. 2018;78:4586–98. 10.1158/0008-5472.Can-17-3841. 29880482 10.1158/0008-5472.CAN-17-3841

[74] Cooks T, Pateras I, Jenkins L, Patel K, Robles A, Morris J, et al. Mutant p53 cancers reprogram macrophages to tumor supporting macrophages via exosomal miR-1246. Nat Commun. 2018;9:771. 10.1038/s41467-018-03224-w. 29472616 10.1038/s41467-018-03224-w PMC5823939

[75] Gerloff D, Lützkendorf J, Moritz R, Wersig T, Mäder K, Müller L, et al. Melanoma-derived exosomal miR-125b-5p educates tumor associated macrophages (TAMs) by targeting lysosomal acid lipase A (LIPA). Cancers. 2020;12:464. 10.3390/cancers12020464. 10.3390/cancers12020464 PMC7072270 32079286

[76] Hao X, Zheng Z, Liu H, Zhang Y, Kang J, Kong X, et al. Inhibition of APOC1 promotes the transformation of M2 into M1 macrophages via the ferroptosis pathway and enhances anti-PD1 immunotherapy in hepatocellular carcinoma based on single-cell RNA sequencing. Redox Biol. 2022;56:102463. 10.1016/j.redox.2022.102463. 36108528 10.1016/j.redox.2022.102463 PMC9482117

[77] Yang Y, Wang Y, Guo L, Gao W, Tang T, Yan M. Interaction between macrophages and ferroptosis. Cell Death Dis. 2022;13:355. 10.1038/s41419-022-04775-z. 35429990 10.1038/s41419-022-04775-z PMC9013379

[78] Ito F, Kato K, Yanatori I, Murohara T, Toyokuni S. Ferroptosis-dependent extracellular vesicles from macrophage contribute to asbestos-induced mesothelial carcinogenesis through loading ferritin. Redox Biol. 2021;47:102174. 10.1016/j.redox.2021.102174. 34700146 10.1016/j.redox.2021.102174 PMC8577498

[79] Wang Y, Fang J, Liu B, Shao C, Shi Y. Reciprocal regulation of mesenchymal stem cells and immune responses. Cell Stem Cell. 2022;29:1515–30. 10.1016/j.stem.2022.10.001. 36332569 10.1016/j.stem.2022.10.001

[80] Ma T, Wu J, Mu J, Gao J. Biomaterials reinforced MSCs transplantation for spinal cord injury repair. Asian J Pharm Sci. 2022;17:4–19. 10.1016/j.ajps.2021.03.003. 35261642 10.1016/j.ajps.2021.03.003 PMC8888140

[81] Su I, Su Y, Setiawan S, Yadav V, Fong I, Yeh C, et al. NADPH oxidase subunit CYBB confers chemotherapy and ferroptosis resistance in mesenchymal glioblastoma via Nrf2/SOD2 modulation. Int J Mol Sci. 2023;24:7706. 10.3390/ijms24097706. 10.3390/ijms24097706 PMC10178261 37175412

[82] Lin Z, Wu Y, Xu Y, Li G, Li Z, Liu T. Mesenchymal stem cell-derived exosomes in cancer therapy resistance: recent advances and therapeutic potential. Mol Cancer. 2022;21:179. 10.1186/s12943-022-01650-5. 36100944 10.1186/s12943-022-01650-5 PMC9468526

[83] Karnoub A, Dash A, Vo A, Sullivan A, Brooks M, Bell G, et al. Mesenchymal stem cells within tumour stroma promote breast cancer metastasis. Nature. 2007;449:557–63. 10.1038/nature06188. 17914389 10.1038/nature06188

[84] Lu J, Dong Q, Zhang S, Feng Y, Yang J, Zhao L. Acute myeloid leukemia (AML)-derived mesenchymal stem cells induce chemoresistance and epithelial-mesenchymal transition-like program in AML through IL-6/JAK2/STAT3 signaling. Cancer Sci. 2023;114:3287–300. 10.1111/cas.15855. 37272257 10.1111/cas.15855 PMC10394133

[85] Li H, Li F. Exosomes from BM-MSCs increase the population of CSCs via transfer of miR-142-3p. Br J Cancer. 2018;119:744–55. 10.1038/s41416-018-0254-z. 30220706 10.1038/s41416-018-0254-z PMC6173771

[86] Lyu T, Wang Y, Li D, Yang H, Qin B, Zhang W, et al. Exosomes from BM-MSCs promote acute myeloid leukemia cell proliferation, invasion and chemoresistance via upregulation of S100A4. Exp Hematol Oncol. 2021;10:24. 10.1186/s40164-021-00220-7. 33789743 10.1186/s40164-021-00220-7 PMC8011411

[87] Zhang L, Khadka B, Wu J, Feng Y, Long B, Xiao R, et al. Bone marrow mesenchymal stem cells-derived exosomal miR-425-5p inhibits acute myeloid leukemia cell proliferation, apoptosis, invasion and migration by targeting WTAP. OncoTargets Ther. 2021;14:4901–14. 10.2147/ott.S286326. 10.2147/OTT.S286326 PMC8478487 34594112

[88] Lin F, Chen W, Zhou J, Zhu J, Yao Q, Feng B, et al. Mesenchymal stem cells protect against ferroptosis via exosome-mediated stabilization of SLC7A11 in acute liver injury. Cell Death Dis. 2022;13:271. 10.1038/s41419-022-04708-w. 35347117 10.1038/s41419-022-04708-w PMC8960810

[89] Wu L, Tian X, Zuo H, Zheng W, Li X, Yuan M, et al. miR-124-3p delivered by exosomes from heme oxygenase-1 modified bone marrow mesenchymal stem cells inhibits ferroptosis to attenuate ischemia-reperfusion injury in steatotic grafts. J Nanobiotechnol. 2022;20:196. 10.1186/s12951-022-01407-8. 10.1186/s12951-022-01407-8 PMC9026664 35459211

[90] Yu Y, Wu T, Lu Y, Zhao W, Zhang J, Chen Q, et al. Exosomal thioredoxin-1 from hypoxic human umbilical cord mesenchymal stem cells inhibits ferroptosis in doxorubicin-induced cardiotoxicity via mTORC1 signaling. Free Radic Biol Med. 2022;193:108–21. 10.1016/j.freeradbiomed.2022.10.268. 36241072 10.1016/j.freeradbiomed.2022.10.268

[91] Chen L, Liu Y, Wang Z, Zhang L, Xu Y, Li Y, et al. Mesenchymal stem cell-derived extracellular vesicles protect against abdominal aortic aneurysm formation by inhibiting NET-induced ferroptosis. Exp Mol Med. 2023;55:939–51. 10.1038/s12276-023-00986-2. 37121969 10.1038/s12276-023-00986-2 PMC10238484

[92] Wei Z, Hang S, Wiredu Ocansey D, Zhang Z, Wang B, Zhang X, et al. Human umbilical cord mesenchymal stem cells derived exosome shuttling mir-129-5p attenuates inflammatory bowel disease by inhibiting ferroptosis. J Nanobiotechnol. 2023;21:188. 10.1186/s12951-023-01951-x. 10.1186/s12951-023-01951-x PMC10259042 37303049

[93] Atiya H, Frisbie L, Goldfeld E, Orellana T, Donnellan N, Modugno F, et al. Endometriosis-associated mesenchymal stem cells support ovarian clear cell carcinoma through iron regulation. Cancer Res. 2022;82:4680–93. 10.1158/0008-5472.Can-22-1294. 36219681 10.1158/0008-5472.CAN-22-1294 PMC9755968

[94] Dai S, Li F, Long H, Zhou Z, Luo H, Xu S, et al. Relationship between miRNA and ferroptosis in tumors. Front Pharmacol. 2022;13:977062. 10.3389/fphar.2022.977062. 36408273 10.3389/fphar.2022.977062 PMC9672467

[95] Zhu L, Sun H, Wang S, Huang S, Zheng Y, Wang C, et al. Isolation and characterization of exosomes for cancer research. J Hematol Oncol. 2020;13:152. 10.1186/s13045-020-00987-y. 33168028 10.1186/s13045-020-00987-y PMC7652679

[96] Chen W, Bao L, Ren Q, Zhang Z, Yi L, Lei W, et al. SCARB1 in extracellular vesicles promotes NPC metastasis by co-regulating M1 and M2 macrophage function. Cell Death Discov. 2023;9:323. 10.1038/s41420-023-01621-9. 37644041 10.1038/s41420-023-01621-9 PMC10465564

[97] Gerstberger S, Jiang Q, Ganesh K. Metastasis. Cell. 2023;186:1564–79. 10.1016/j.cell.2023.03.003. 37059065 10.1016/j.cell.2023.03.003 PMC10511214

[98] Hoshino A, Costa-Silva B, Shen T, Rodrigues G, Hashimoto A, Tesic Mark M, et al. Tumour exosome integrins determine organotropic metastasis. Nature. 2015;527:329–35. 10.1038/nature15756. 26524530 10.1038/nature15756 PMC4788391

[99] Qi M, Xia Y, Wu Y, Zhang Z, Wang X, Lu L, et al. Lin28B-high breast cancer cells promote immune suppression in the lung pre-metastatic niche via exosomes and support cancer progression. Nat Commun. 2022;13:897. 10.1038/s41467-022-28438-x. 35173168 10.1038/s41467-022-28438-x PMC8850492

[100] Zeng Z, Li Y, Pan Y, Lan X, Song F, Sun J, et al. Cancer-derived exosomal miR-25-3p promotes pre-metastatic niche formation by inducing vascular permeability and angiogenesis. Nat Commun. 2018;9:5395. 10.1038/s41467-018-07810-w. 30568162 10.1038/s41467-018-07810-w PMC6300604

[101] Yuan X, Qian N, Ling S, Li Y, Sun W, Li J, et al. Breast cancer exosomes contribute to pre-metastatic niche formation and promote bone metastasis of tumor cells. Theranostics. 2021;11:1429–45. 10.7150/thno.45351. 33391543 10.7150/thno.45351 PMC7738874

[102] Feng W, Dean D, Hornicek F, Shi H, Duan Z. Exosomes promote pre-metastatic niche formation in ovarian cancer. Mol Cancer. 2019;18:124. 10.1186/s12943-019-1049-4. 31409361 10.1186/s12943-019-1049-4 PMC6691526

[103] Morrissey S, Zhang F, Ding C, Montoya-Durango D, Hu X, Yang C, et al. Tumor-derived exosomes drive immunosuppressive macrophages in a pre-metastatic niche through glycolytic dominant metabolic reprogramming. Cell Metab. 2021;33:2040–58.e10. 10.1016/j.cmet.2021.09.002. 34559989 10.1016/j.cmet.2021.09.002 PMC8506837

[104] Lobb R, Lima L, Möller A. Exosomes: key mediators of metastasis and pre-metastatic niche formation. Semin Cell Dev Biol. 2017;67:3–10. 10.1016/j.semcdb.2017.01.004. 28077297 10.1016/j.semcdb.2017.01.004

[105] Guo Y, Ji X, Liu J, Fan D, Zhou Q, Chen C, et al. Effects of exosomes on pre-metastatic niche formation in tumors. Mol Cancer. 2019;18:39. 10.1186/s12943-019-0995-1. 30857545 10.1186/s12943-019-0995-1 PMC6413442

[106] He G, Peng X, Wei S, Yang S, Li X, Huang M, et al. Exosomes in the hypoxic TME: from release, uptake and biofunctions to clinical applications. Mol Cancer. 2022;21:19. 10.1186/s12943-021-01440-5. 35039054 10.1186/s12943-021-01440-5 PMC8762953

[107] Chen W, Zuo F, Zhang K, Xia T, Lei W, Zhang Z, et al. Exosomal MIF derived from nasopharyngeal carcinoma promotes metastasis by repressing ferroptosis of macrophages. Front Cell Dev Biol. 2021;9:791187. 10.3389/fcell.2021.791187. 35036405 10.3389/fcell.2021.791187 PMC8758577

[108] Li F, Xu T, Chen P, Sun R, Li C, Zhao X, et al. Platelet-derived extracellular vesicles inhibit ferroptosis and promote distant metastasis of nasopharyngeal carcinoma by upregulating ITGB3. Int J Biol Sci. 2022;18:5858–72. 10.7150/ijbs.76162. 36263165 10.7150/ijbs.76162 PMC9576525

[109] Huang J, Pan H, Sun J, Wu J, Xuan Q, Wang J, et al. TMEM147 aggravates the progression of HCC by modulating cholesterol homeostasis, suppressing ferroptosis, and promoting the M2 polarization of tumor-associated macrophages. J Exp Clin Cancer Res. 2023;42:286. 10.1186/s13046-023-02865-0. 37891677 10.1186/s13046-023-02865-0 PMC10612308

[110] Xu C, Sun S, Johnson T, Qi R, Zhang S, Zhang J, et al. The glutathione peroxidase Gpx4 prevents lipid peroxidation and ferroptosis to sustain Treg cell activation and suppression of antitumor immunity. Cell Rep. 2021;35:109235. 10.1016/j.celrep.2021.109235. 34133924 10.1016/j.celrep.2021.109235

[111] Zhao Y, Liu Z, Liu G, Zhang Y, Liu S, Gan D, et al. Neutrophils resist ferroptosis and promote breast cancer metastasis through aconitate decarboxylase 1. Cell Metab. 2023;35:1688–703.e10. 10.1016/j.cmet.2023.09.004. 37793345 10.1016/j.cmet.2023.09.004 PMC10558089

[112] Wiernicki B, Maschalidi S, Pinney J, Adjemian S, Vanden Berghe T, Ravichandran K, et al. Cancer cells dying from ferroptosis impede dendritic cell-mediated anti-tumor immunity. Nat Commun. 2022;13:3676. 10.1038/s41467-022-31218-2. 35760796 10.1038/s41467-022-31218-2 PMC9237053

[113] Wang W, Green M, Choi J, Gijón M, Kennedy P, Johnson J, et al. CD8 T cells regulate tumour ferroptosis during cancer immunotherapy. Nature. 2019;569:270–4. 10.1038/s41586-019-1170-y. 31043744 10.1038/s41586-019-1170-y PMC6533917

[114] Kim R, Hashimoto A, Markosyan N, Tyurin V, Tyurina Y, Kar G, et al. Ferroptosis of tumour neutrophils causes immune suppression in cancer. Nature. 2022;612:338–46. 10.1038/s41586-022-05443-0. 36385526 10.1038/s41586-022-05443-0 PMC9875862

[115] Huang Y, Wang S, Ke A, Guo K. Ferroptosis and its interaction with tumor immune microenvironment in liver cancer. Biochim et Biophys Acta Rev Cancer. 2023;1878:188848. 10.1016/j.bbcan.2022.188848. 10.1016/j.bbcan.2022.188848 36502929

[116] Jin P, Jiang J, Zhou L, Huang Z, Nice E, Huang C, et al. Mitochondrial adaptation in cancer drug resistance: prevalence, mechanisms, and management. J Hematol Oncol. 2022;15:97. 10.1186/s13045-022-01313-4. 35851420 10.1186/s13045-022-01313-4 PMC9290242

[117] Song H, Liu D, Dong S, Zeng L, Wu Z, Zhao P, et al. Epitranscriptomics and epiproteomics in cancer drug resistance: therapeutic implications. Signal Transduct Target Ther. 2020;5:193. 10.1038/s41392-020-00300-w. 32900991 10.1038/s41392-020-00300-w PMC7479143

[118] Gao L, Wu Z, Assaraf Y, Chen Z, Wang L. Overcoming anti-cancer drug resistance via restoration of tumor suppressor gene function. Drug Resist Updates. 2021;57:100770. 10.1016/j.drup.2021.100770. 10.1016/j.drup.2021.100770 34175687

[119] Gottesman M. Mechanisms of cancer drug resistance. Annu Rev Med. 2002;53:615–27. 10.1146/annurev.med.53.082901.103929. 11818492 10.1146/annurev.med.53.082901.103929

[120] Liu Z, Zou H, Dang Q, Xu H, Liu L, Zhang Y, et al. Biological and pharmacological roles of mA modifications in cancer drug resistance. Mol Cancer. 2022;21:220. 10.1186/s12943-022-01680-z. 36517820 10.1186/s12943-022-01680-z PMC9749187

[121] Qu X, Liu B, Wang L, Liu L, Zhao W, Liu C, et al. Loss of cancer-associated fibroblast-derived exosomal DACT3-AS1 promotes malignant transformation and ferroptosis-mediated oxaliplatin resistance in gastric cancer. Drug Resist Updates. 2023;68:100936. 10.1016/j.drup.2023.100936. 10.1016/j.drup.2023.100936 36764075

[122] Zhang Y, Liu X, Zeng L, Zhao X, Chen Q, Pan Y, et al. Exosomal protein angiopoietin-like 4 mediated radioresistance of lung cancer by inhibiting ferroptosis under hypoxic microenvironment. Br J Cancer. 2022;127:1760–72. 10.1038/s41416-022-01956-7. 36050447 10.1038/s41416-022-01956-7 PMC9643351

[123] Yang Y, Gu H, Zhang K, Guo Z, Wang X, Wei Q, et al. Exosomal ACADM sensitizes gemcitabine-resistance through modulating fatty acid metabolism and ferroptosis in pancreatic cancer. BMC Cancer. 2023;23:789. 10.1186/s12885-023-11239-w. 37612627 10.1186/s12885-023-11239-w PMC10463774

[124] Song Z, Jia G, Ma P, Cang S. Exosomal miR-4443 promotes cisplatin resistance in non-small cell lung carcinoma by regulating FSP1 m6A modification-mediated ferroptosis. Life Sci. 2021;276:119399. 10.1016/j.lfs.2021.119399. 33781830 10.1016/j.lfs.2021.119399

[125] Tao J, Yang G, Zhou W, Qiu J, Chen G, Luo W, et al. Targeting hypoxic tumor microenvironment in pancreatic cancer. J Hematol Oncol. 2021;14:14. 10.1186/s13045-020-01030-w. 33436044 10.1186/s13045-020-01030-w PMC7805044

[126] Chen Z, Han F, Du Y, Shi H, Zhou W. Hypoxic microenvironment in cancer: molecular mechanisms and therapeutic interventions. Signal Transduct Target Ther. 2023;8:70. 10.1038/s41392-023-01332-8. 36797231 10.1038/s41392-023-01332-8 PMC9935926

[127] Wang D, Qiu G, Zhu X, Wang Q, Zhu C, Fang C, et al. Macrophage-inherited exosome excise tumor immunosuppression to expedite immune-activated ferroptosis. J Immunother Cancer. 2023;11:e006516. 10.1136/jitc-2022-006516. 10.1136/jitc-2022-006516 PMC10193064 37192783

[128] Gao Y, Huang Y, Zhao Y, Hu P. Cancer-associated fibroblast-secreted exosomal miR-454-3p inhibits lipid metabolism and ferroptosis in breast cancer by targeting ACSL4. Naunyn-Schmiedeberg’s Archiv Pharmacol. 2024. 10.1007/s00210-024-03488-8. 10.1007/s00210-024-03488-8 39373750

[129] Yang J, Zhang M, Zhang X, Zhou Y, Ma T, Liang J, et al. Glioblastoma-derived exosomes promote lipid accumulation and induce ferroptosis in dendritic cells via the NRF2/GPX4 pathway. Front Immunol. 2024;15:1439191. 10.3389/fimmu.2024.1439191. 39192971 10.3389/fimmu.2024.1439191 PMC11347305

[130] Zhou B, Xu K, Zheng X, Chen T, Wang J, Song Y, et al. Application of exosomes as liquid biopsy in clinical diagnosis. Signal Transduct Target Ther. 2020;5:144. 10.1038/s41392-020-00258-9. 32747657 10.1038/s41392-020-00258-9 PMC7400738

[131] Yu D, Li Y, Wang M, Gu J, Xu W, Cai H, et al. Exosomes as a new frontier of cancer liquid biopsy. Mol Cancer. 2022;21:56. 10.1186/s12943-022-01509-9. 35180868 10.1186/s12943-022-01509-9 PMC8855550

[132] Wang Y, Chen Q, Song H, Zhang Y, Chen H, Liu P, et al. A triple therapeutic strategy with antiexosomal iron efflux for enhanced ferroptosis therapy and immunotherapy. Small. 2022;18:e2201704. 10.1002/smll.202201704. 36071027 10.1002/smll.202201704

[133] Liang Y, Duan L, Lu J, Xia J. Engineering exosomes for targeted drug delivery. Theranostics. 2021;11:3183–95. 10.7150/thno.52570. 33537081 10.7150/thno.52570 PMC7847680

[134] Zhang M, Hu S, Liu L, Dang P, Liu Y, Sun Z, et al. Engineered exosomes from different sources for cancer-targeted therapy. Signal Transduct Target Ther. 2023;8:124 10.1038/s41392-023-01382-y. 36922504 10.1038/s41392-023-01382-y PMC10017761

[135] Yu M, Gai C, Li Z, Ding D, Zheng J, Zhang W, et al. Targeted exosome-encapsulated erastin induced ferroptosis in triple negative breast cancer cells. Cancer Sci. 2019;110:3173–82. 10.1111/cas.14181. 31464035 10.1111/cas.14181 PMC6778638

[136] Chen W, Li Z, Yu N, Zhang L, Li H, Chen Y, et al. Bone-targeting exosome nanoparticles activate Keap1/Nrf2/GPX4 signaling pathway to induce ferroptosis in osteosarcoma cells. J Nanobiotechnol. 2023;21:355. 10.1186/s12951-023-02129-1. 10.1186/s12951-023-02129-1 PMC10541697 37775799

[137] Du J, Wan Z, Wang C, Lu F, Wei M, Wang D, et al. Designer exosomes for targeted and efficient ferroptosis induction in cancer via chemo-photodynamic therapy. Theranostics. 2021;11:8185–96. 10.7150/thno.59121. 34373736 10.7150/thno.59121 PMC8344009

[138] Parada N, Romero-Trujillo A, Georges N, Alcayaga-Miranda F. Camouflage strategies for therapeutic exosomes evasion from phagocytosis. J Adv Res. 2021;31:61–74. 10.1016/j.jare.2021.01.001. 34194832 10.1016/j.jare.2021.01.001 PMC8240105

[139] Li B, Chen X, Qiu W, Zhao R, Duan J, Zhang S, et al. Synchronous disintegration of ferroptosis defense axis via engineered exosome-conjugated magnetic nanoparticles for glioblastoma therapy. Adv Sci. 2022;9:e2105451 10.1002/advs.202105451. 10.1002/advs.202105451 PMC9189685 35508804

[140] Katz O, Shaked Y. Host effects contributing to cancer therapy resistance. Drug Resist Updates. 2015;19:33–42. 10.1016/j.drup.2014.12.002. 10.1016/j.drup.2014.12.002 25575621

[141] Thakur A, Sidu R, Zou H, Alam M, Yang M, Lee Y. Inhibition of glioma cells’ proliferation by doxorubicin-loaded exosomes via microfluidics. Int J Nanomed. 2020;15:8331–43. 10.2147/ijn.S263956. 10.2147/IJN.S263956 PMC7605152 33149579

[142] Tian Y, Li S, Song J, Ji T, Zhu M, Anderson G, et al. A doxorubicin delivery platform using engineered natural membrane vesicle exosomes for targeted tumor therapy. Biomaterials. 2014;35:2383–90. 10.1016/j.biomaterials.2013.11.083. 24345736 10.1016/j.biomaterials.2013.11.083

[143] Tian C, Yang Y, Bai B, Wang S, Liu M, Sun R, et al. Potential of exosomes as diagnostic biomarkers and therapeutic carriers for doxorubicin-induced cardiotoxicity. Int J Biol Sci. 2021;17:1328–38. 10.7150/ijbs.58786. 33867849 10.7150/ijbs.58786 PMC8040474

[144] Sun J, Liu Q, Jiang Y, Cai Z, Liu H, Zuo H. Engineered small extracellular vesicles loaded with miR-654-5p promote ferroptosis by targeting HSPB1 to alleviate sorafenib resistance in hepatocellular carcinoma. Cell Death Discov. 2023;9:362. 10.1038/s41420-023-01660-2. 37777559 10.1038/s41420-023-01660-2 PMC10542782

[145] Li X, Yu Q, Zhao R, Guo X, Liu C, Zhang K, et al. Designer exosomes for targeted delivery of a novel therapeutic cargo to enhance sorafenib-mediated ferroptosis in hepatocellular carcinoma. Front Oncol. 2022;12:898156. 10.3389/fonc.2022.898156. 35814401 10.3389/fonc.2022.898156 PMC9263838

[146] Xie Q, Zheng J, Ding J, Wu Y, Liu L, Yu Z, et al. Exosome-mediated immunosuppression in tumor microenvironments. Cells. 2022;11:1946. 10.3390/cells11121946. 10.3390/cells11121946 PMC9221707 35741075

[147] Cheng L, Zhang P, Liu Y, Liu Z, Tang J, Xu L, et al. Multifunctional hybrid exosomes enhanced cancer chemo-immunotherapy by activating the STING pathway. Biomaterials. 2023;301:122259. 10.1016/j.biomaterials.2023.122259. 37531777 10.1016/j.biomaterials.2023.122259

[148] Poggio M, Hu T, Pai C, Chu B, Belair C, Chang A, et al. Suppression of exosomal PD-L1 induces systemic anti-tumor immunity and memory. Cell. 2019;177:414–27.e13. 10.1016/j.cell.2019.02.016. 30951669 10.1016/j.cell.2019.02.016 PMC6499401

[149] Wang G, Xie L, Li B, Sang W, Yan J, Li J, et al. A nanounit strategy reverses immune suppression of exosomal PD-L1 and is associated with enhanced ferroptosis. Nat Commun. 2021;12:5733. 10.1038/s41467-021-25990-w. 34593794 10.1038/s41467-021-25990-w PMC8484261

[150] Xie L, Li J, Wang G, Sang W, Xu M, Li W, et al. Phototheranostic metal-phenolic networks with antiexosomal PD-L1 enhanced ferroptosis for synergistic immunotherapy. J Am Chem Soc. 2022;144:787–97. 10.1021/jacs.1c09753. 34985903 10.1021/jacs.1c09753

[151] Liu N, Chen M. Crosstalk between ferroptosis and cuproptosis: from mechanism to potential clinical application. Biomed Pharmacother = Biomed Pharmacother. 2024;171:116115. 10.1016/j.biopha.2023.116115. 38181713 10.1016/j.biopha.2023.116115

